# Toxicogenomic Characterization of Common Genes and Signaling Pathways Associated with Multiple Toxic Metal(loid)s in Lung Cancer: A Comparative Analysis of Small-Cell and Non-Small-Cell Lung Cancer

**DOI:** 10.3390/cancers18152395

**Published:** 2026-07-25

**Authors:** Katarina Živančević, Kıvanç Kök

**Affiliations:** 1 Department of General Physiology and Biophysics, Center for Laser Microscopy, Institute of Physiology and Biochemistry “Ivan Djaja”, Faculty of Biology, University of Belgrade, Studentski trg 16, 11158 Belgrade, Serbia; 2 Research Institute for Health Sciences and Technologies (SABITA), Istanbul Medipol University, Istanbul 34810, Türkiye; guvanch@medipol.edu.tr; 3 Department of Biostatistics and Medical Informatics, International School of Medicine, Istanbul Medipol University, Istanbul 34810, Türkiye

**Keywords:** toxic metal(loid)s, lung cancer, non-small cell lung cancer, small cell lung cancer, toxicogenomics, TP53, PI3K–AKT–mTOR signaling, VEGF signaling, MAPK signaling, Comparative Toxicogenomics Database

## Abstract

**Background/Objectives**: This study aimed to identify common target genes, molecular interaction networks, and signaling pathways shared across the investigated toxic metal(loid)s—lead (Pb), cadmium (Cd), methylmercury compounds (MMC), arsenic (As), nickel (Ni), and hexavalent chromium (Cr(VI))—and associated with lung cancer overall, non-small-cell lung cancer (NSCLC), and small-cell lung cancer (SCLC). **Methods**: The Comparative Toxicogenomics Database (CTD) was used to identify associated genes, GeneMANIA to characterize molecular interaction networks, and ToppFun for pathway enrichment analysis. **Results**: Two common genes (*MTOR* and *TP53*) were identified for SCLC, eight for NSCLC (*CAT*, *CCNB1*, *MTOR*, *NQO1*, *SOD2*, *STAT3*, *TP53*, and *VEGFA*), and eighteen for lung cancer overall. Gene network analysis revealed predominantly physical interactions in SCLC, pathway interactions in NSCLC, and co-expression in lung cancer overall. Several toxic metal(loid)s exhibited concordant effects on TP53, VEGFA, HMOX1, and IL6 expression. *TP53* was the only gene common to all investigated toxic metal(loid)s and lung cancer categories. Pathway enrichment identified PI3K–AKT–mTOR signaling in both SCLC and NSCLC, VEGF signaling in NSCLC, and MAPK, interleukin, and macrophage-stimulating protein (MSP) signaling in lung cancer overall. **Conclusions**: The identified genes and pathways suggest molecular mechanisms potentially relevant to the association between exposure to the investigated toxic metal(loid)s and lung carcinogenesis, particularly those related to cell survival, inflammation, oxidative stress, and angiogenesis. *TP53* may represent a common molecular link warranting further investigation. The identified pathways represent candidate biomarkers and mechanistic targets, highlighting the biological heterogeneity of lung cancer and the importance of subtype-specific analyses.

## Simple Summary

Exposure to toxic metals and metalloids has been associated with an increased risk of lung cancer, yet the underlying molecular mechanisms remain incompletely understood, particularly in the context of combined environmental exposure. In this study, we used publicly available toxicogenomic databases and bioinformatics tools to identify genes, interaction networks, and signaling pathways commonly associated with the investigated toxic metal(loid)s (lead, cadmium, methylmercury, arsenic, nickel, and hexavalent chromium) and lung carcinogenesis. We identified several genes and signaling pathways implicated in biological processes relevant to lung cancer, including cell survival, inflammation, oxidative stress, and angiogenesis. TP53 was consistently identified across all investigated lung cancer categories, suggesting its relevance as a common molecular link warranting further investigation. Predicted molecular interaction networks and enriched pathways differed between lung cancer subtypes, underscoring the biological heterogeneity of the disease. These findings generate hypotheses and identify candidate biomarkers for future experimental, epidemiological, and mechanistic studies of combined toxic metal(loid) exposure.

## 1. Introduction

Lung cancer remains one of the most significant public health challenges worldwide, continuing to impose a substantial burden on healthcare systems despite advances in prevention, diagnosis, and treatment. Demographic projections suggest that the annual number of new cancer cases could rise to 35 million by 2050, representing a 77% increase compared with 2022 levels, emphasizing the growing urgency of effective cancer control strategies. Within this context, lung cancer stands out as the most frequently diagnosed cancer and the leading cause of cancer-related mortality globally. In 2022, it accounted for nearly 2.5 million new cases (approximately one in every eight cancers diagnosed worldwide) and was responsible for an estimated 1.8 million deaths, highlighting its profound impact on global health and reflecting marked geographic, socioeconomic, and sex-related disparities in disease burden [1]. The disease is broadly categorized into non-small-cell lung cancer (NSCLC), which comprises approximately 85% of cases and includes adenocarcinoma, squamous cell cancer, and large-cell cancer, and small-cell lung cancer (SCLC), a more aggressive subtype characterized by rapid growth and early metastasis [2]. Although cigarette smoking remains the predominant risk factor, lung cancer is increasingly recognized as a multifactorial disease influenced by occupational exposures, radon, air pollution, and toxic metal(loid)s such as arsenic (As), cadmium (Cd), nickel (Ni), chromium (Cr), lead (Pb) and mercury (Hg) [2,3,4].

Although these toxic metal(loid)s occur naturally in the environment, industrial activities and other anthropogenic sources have substantially increased their environmental burden [5]. Consequently, they are widely distributed in air, water, and soil, where their persistence, bioaccumulative potential, and long biological half-lives contribute to sustained human exposure. Importantly, exposure most often occurs through mixtures rather than individual metal(loid)s, thereby increasing the complexity of their biological and toxicological effects [5]. Despite regulatory efforts to limit environmental contamination, exposure to Pb, Cd, Hg, As, Ni, and Cr remains a significant public health concern [6]. Human exposure continues to occur through contaminated food and water, airborne particles, consumer products, and tobacco smoke [6].

According to the International Agency for Research on Cancer (IARC), arsenic (As), cadmium (Cd), nickel (Ni), and hexavalent chromium (Cr(VI)) are recognized as Group 1 carcinogens, indicating established carcinogenicity in humans [7]. Lead (Pb) is categorized as Group 2A (probably carcinogenic to humans), supported by evidence from occupational exposure studies linking prolonged lead exposure with elevated risks of lung cancer and other malignancies. Although elemental mercury and inorganic mercury compounds are not currently classified as human carcinogens and remain within IARC Group 3, growing evidence raises concerns regarding their potential indirect genotoxic mechanisms [7]. By contrast, methylmercury compounds (MMCs) are classified as Group 2B (possibly carcinogenic to humans), reflecting sufficient evidence of carcinogenicity in experimental animals but limited evidence in human populations [7,8]. Their toxic effects are mediated through diverse molecular and cellular mechanisms, including binding to protein functional groups, interference with essential elements, induction of oxidative stress, epigenetic modifications, inflammatory responses, and apoptosis [4,9]. Additionally, the importance of Pb, Cd, Hg, and As to public health is underscored by their classification by the World Health Organization (WHO) as substances of major concern and by their inclusion among the highest-priority hazardous substances identified by the Agency for Toxic Substances and Disease Registry (ATSDR) [10,11].

Recent advances in computational biology and in silico analyses have accelerated the identification of molecular and epigenetic biomarkers, including DNA methylation signatures, microRNAs, and gene-expression profiles, offering promising tools for early diagnosis and personalized treatment strategies. Moreover, growing evidence suggests that exposure to environmental metal mixtures may contribute to lung carcinogenesis through mechanisms involving oxidative stress, inflammation, and epigenetic dysregulation, further underscoring the importance of integrating exposure assessment with biomarker discovery to better understand lung cancer susceptibility and progression [12].

Accordingly, the present study addresses current toxicological challenges by integrating publicly available toxicogenomic data to identify genes, molecular interaction networks, and signaling pathways shared across the investigated toxic metal(loid)s (Pb, Cd, MMC, As, Ni and Cr(VI)) and lung cancer, with the following objectives:

1. To identify common target genes and characterize their regulatory alterations, molecular networks, and signaling pathways potentially associated with the investigated toxic metal(loid)s and lung carcinogenesis, while elucidating subtype-specific molecular mechanisms relevant to NSCLC and SCLC.
2. To compare the toxicogenomic associations of the individual investigated toxic metal(loid)s and identify common molecular mediators and candidate biomarkers associated with metal(loid)-related lung carcinogenesis, including potential subtype-specific mechanisms relevant to NSCLC and SCLC.
3. To generate mechanistically informed hypotheses regarding molecular mechanisms potentially underlying the association between exposure to the investigated toxic metal(loid)s (Pb, Cd, MMC, As, Ni, and Cr(VI)) and lung carcinogenesis, including NSCLC and SCLC, through the integration of toxicogenomic data, gene interaction networks, and pathway enrichment analyses.

## 2. Materials and Methods

### 2.1. In Silico Analysis

**Figure 1:**
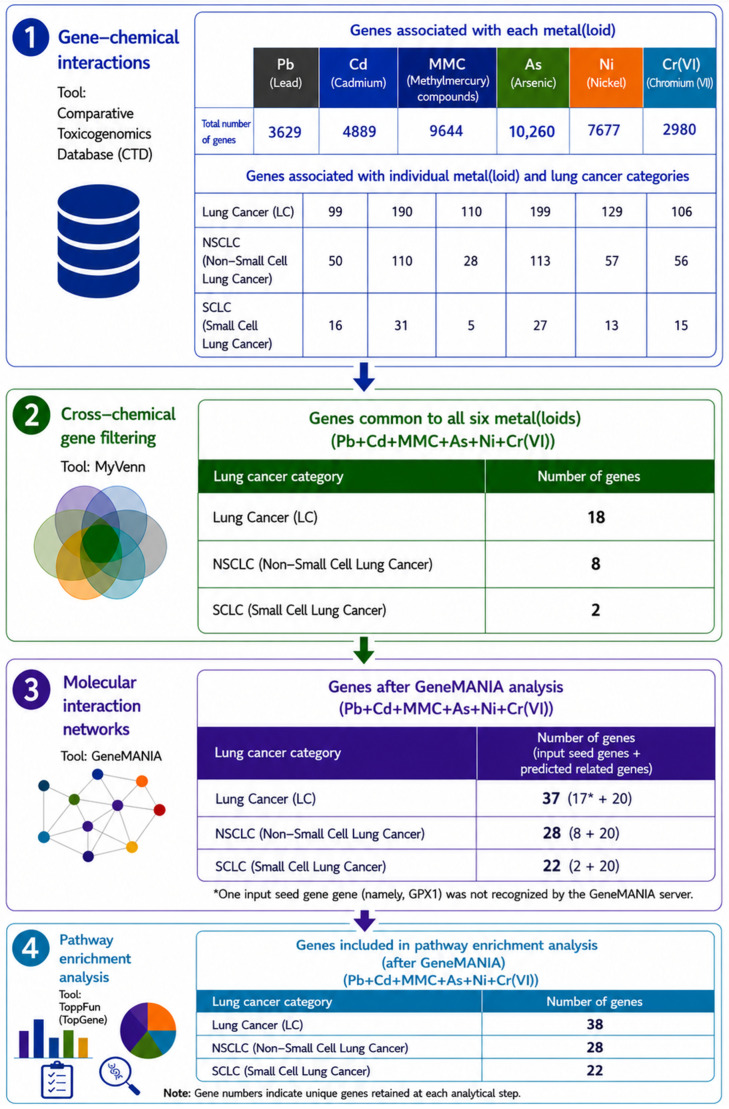
Workflow of the in silico analyses performed in this study.

The workflow of the in silico analyses performed in this study is presented in Figure 1.

#### 2.1.1. Analysis of Data from the Comparative Toxicogenomics Database (CTD)

The Comparative Toxicogenomics Database (North Carolina State University, Raleigh, NC, USA) is a publicly accessible database that provides manually curated information on the relationships among chemical substances, genes, and diseases [13]. To elucidate molecular mechanisms potentially linking the investigated toxic metal(loid)s (Pb, Cd, MMC, As, Ni, and Cr(VI)) with lung carcinogenesis, including subtype-specific mechanisms relevant to NSCLC and SCLC, publicly available data from the CTD were analyzed [13] (http://ctdbase.org/) (accessed on 30 June 2026). As the CTD is continuously updated and does not use conventional version numbers for its public web interface, the database access date and corresponding Changelog are reported to ensure reproducibility. According to the CTD Changelog (https://ctdbase.org/about/changes/) (accessed on 30 June 2026), the data used in the final analyses correspond to the database state following the 1 June 2026 update (Changelog 18280).

This in silico analysis included “Lung Cancer”, as an umbrella CTD disease term that encompasses multiple types of lung cancer found in the CTD, and “Non-Small-Cell Lung Cancer” and “Small Cell Lung Cancer” as subtype-specific disease categories. To ensure that no relevant genes were overlooked, gene retrieval was performed in three complementary steps for each investigated toxic metal(loid) (Pb, Cd, MMC, As, Ni, and Cr(VI)) and each lung cancer category (lung cancer as an umbrella CTD disease term, NSCLC and SCLC):

1. Within the Disease tab of the CTD, each lung cancer category was queried using each individual toxic metal(loid) as the chemical of interest, and the associated genes were retrieved.
2. Within the Chemical tab of the CTD, each investigated toxic metal(loid) was queried using each lung cancer category as the disease of interest, and the associated genes were retrieved.
3. The gene lists obtained from both searches were merged, and duplicate records were removed.

All genes included in the analysis were obtained from the CTD Inference Network. In the CTD, chemical–gene interactions presented in the Interaction section are manually curated by CTD biocurators from the published literature. These interactions may be supported by either direct evidence or inferred evidence. To comprehensively capture all genes linking each investigated toxic metal(loid) with each lung cancer category, all inference network genes were included irrespective of whether the underlying curated interactions were based on direct or inferred evidence. No additional evidence-strength thresholds or filtering criteria were applied.

This approach enabled the identification of the total number of genes interacting with the analyzed toxic metal(loid)s, as well as the compilation of gene lists associated with simultaneous exposure to Pb, Cd, MMC, As, Ni, and Cr(VI) and different types of lung cancer. Subsequently, the CTD MyVenn tool (North Carolina State University, Raleigh, NC, USA (https://ctdbase.org/tools/myVenn.go, accessed on 30 June 2026) was used to identify target genes shared between these toxic metal(loid)s and the development of lung cancer, as well as NSCLC and SCLC.

Furthermore, the effects of the investigated toxic metal(loid)s on the identified target genes were evaluated using the “Chemical Interactions” section of the CTD. Only binary chemical–gene interactions reported for human organism (*Homo sapiens*) were included in the analysis. Binary interactions represent manually curated relationships between a single chemical and a single gene (or gene product) and were selected because they enable consistent comparison of the individual toxicogenomic associations of each investigated toxic metal(loid), facilitate identification of shared and distinct molecular responses across the investigated toxic metal(loid)s, and avoid multiple counting of the same biological effect that may occur in more complex interaction records. The analysis was restricted to interactions affecting protein expression or mRNA expression. Complex interactions involving multiple chemicals acting simultaneously on a single gene were not included. When the majority or all investigated metal(loid)s were reported to produce the same effect on gene or protein expression, these findings were interpreted as concordant effects, indicating a common pattern of regulation. Conversely, when different metal(loid)s showed opposing effects on the same gene, these findings were interpreted as divergent or opposing effects.

Methylmercury compounds (MMC) and hexavalent chromium [Cr(VI)] were specifically included in the analysis due to their well-documented carcinogenic properties and higher toxicological significance relative to other forms of mercury and chromium.

#### 2.1.2. GeneMANIA Analysis

The publicly available GeneMANIA prediction server (University of Toronto, Toronto, ON, Canada) [14] (http://genemania.org/) was utilized as an in silico platform for the identification of genes related to the input gene set and the characterization of their interaction profiles (accessed on 30 June 2026). Genes identified by CTD analysis as common to Pb, Cd, MMC, As, Ni, Cr(VI), and lung cancer, as well as NSCLC and SCLC, were uploaded to the server. The default GeneMANIA settings were used for all analyses, including the default output of 20 related genes. This setting was retained to ensure methodological consistency and comparability across all analyses. The resulting interaction networks were subsequently analyzed according to the type of interactions among the identified genes. The GPX1 gene was not recognized by the GeneMANIA server and was therefore excluded from the network analysis. Additional network visualization was performed using Cytoscape version 3.8.0 with the GeneMANIA plug-in (http://genemania.org/plugin/) (accessed on 17 July 2026).

#### 2.1.3. Pathway Enrichment Analysis Based on Functional Annotation Databases

The ToppFun function available within the ToppGene portal (Division of Biomedical Informatics, Cincinnati Children’s Hospital Medical Center, Cincinnati, OH, USA) [15] (https://toppgene.cchmc.org/) was utilized to perform pathway enrichment analysis based on functional annotation databases (accessed on 30 June 2026). This platform integrates biological processes and signaling pathways associated with both exposure to the investigated toxic metal(loid)s and disease progression [15]. Target genes identified through CTD analysis and the MyVenn tool as being associated with lung cancer, as well as NSCLC and SCLC, and exposure to the examined metal(loid)s were used to computationally reconstruct the related gene association networks using GeneMANIA. The gene lists of the resulting networks were submitted to the platform. Subsequently, signaling pathways potentially involved in disease development through shared genes were analyzed. To this end, pathways significantly associated with the input genes were delineated by using ToppFun across the REACTOME, KEGG, WikiPathways (WP), and PID databases. Statistical significance was assessed using the hypergeometric test with Benjamini–Hochberg (BH) false discovery rate (FDR) correction for multiple testing and a *p*-value cutoff of 0.05. Among the significantly enriched pathways, the five most statistically significant pathways, ranked according to the lowest FDR-adjusted *p*-values, were selected for subsequent evaluation. Data regarding the number of genes involved in each selected pathway were also extracted.

## 3. Results

### 3.1. Extraction of Target Genes

**Figure 2:**
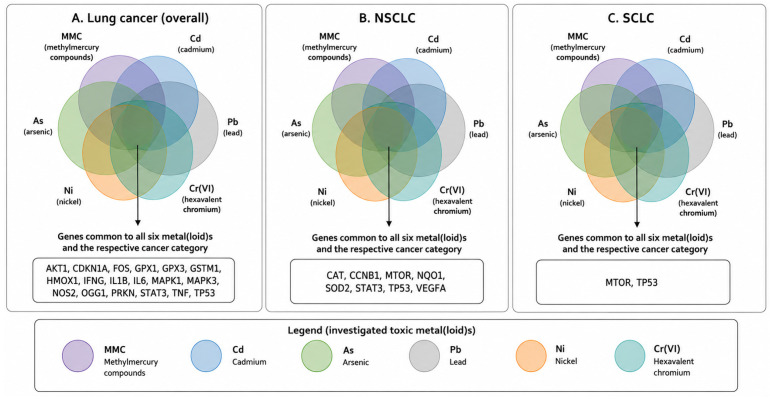
Common target genes associated with Pb, Cd, MMC, As, Ni and Cr(VI) exposure and (**A**) lung cancer, (**B**) non-small-cell lung cancer (NSCLC) and (**C**) small-cell lung cancer (SCLC).

The CTD was examined to determine the overall number of genes interacting with the investigated toxic metal(loid)s. The relationships between exposure to individual toxic metal(loid)s and lung cancer, as well as NSCLC and SCLC, were found in the “Disease” section and are shown in Figure 2 and Supplementary Table S1. The sets of genes common to each investigated lung cancer category and shared across the investigated toxic metal(loid)s (Pb, Cd, MMC, As, Ni, and Cr(VI)) were subsequently identified using the MyVenn tool. In the broader category of “lung cancer,” eighteen genes were identified, including *AKT1*, *CDKN1A*, *FOS*, *GPX1*, *GPX3*, *GSTM1*, *HMOX1*, *IFNG*, *IL1B*, *IL6*, *MAPK1*, *MAPK3*, *NOS2*, *OGG1*, *PRKN*, *STAT3*, *TNF*, and *TP53* (Figure 2 and Supplementary Table S1). In NSCLC, eight genes were identified: *CAT*, *CCNB1*, *MTOR*, *NQO1*, *SOD2*, *STAT3*, *TP53*, and *VEGFA* (Figure 2 and Supplementary Table S1). Two genes, *MTOR* and *TP53*, were identified as common interacting genes associated with SCLC and shared across the investigated toxic metal(loid)s (Pb, Cd, MMC, As, Ni, and Cr(VI)) (Figure 2, Supplementary Figures S1–S3 and Supplementary Table S1).

### 3.2. Identification of the Type of Interactions Between the Target Genes

**Figure 3:**
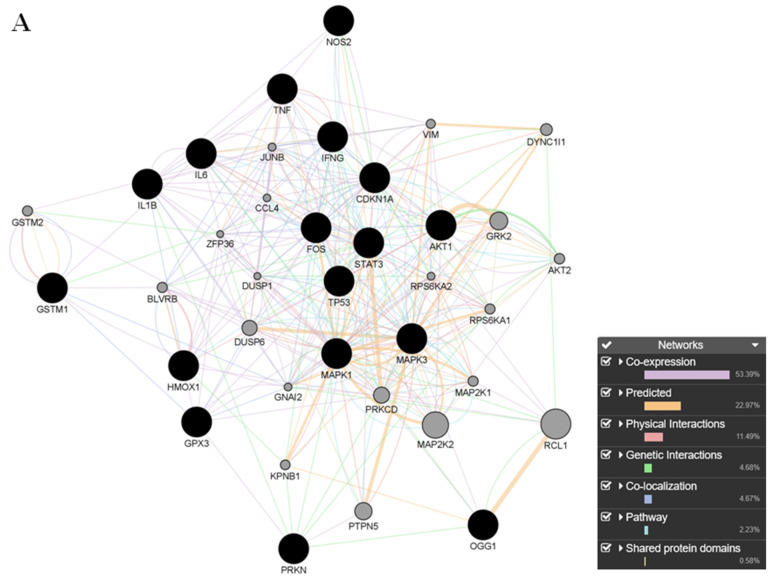

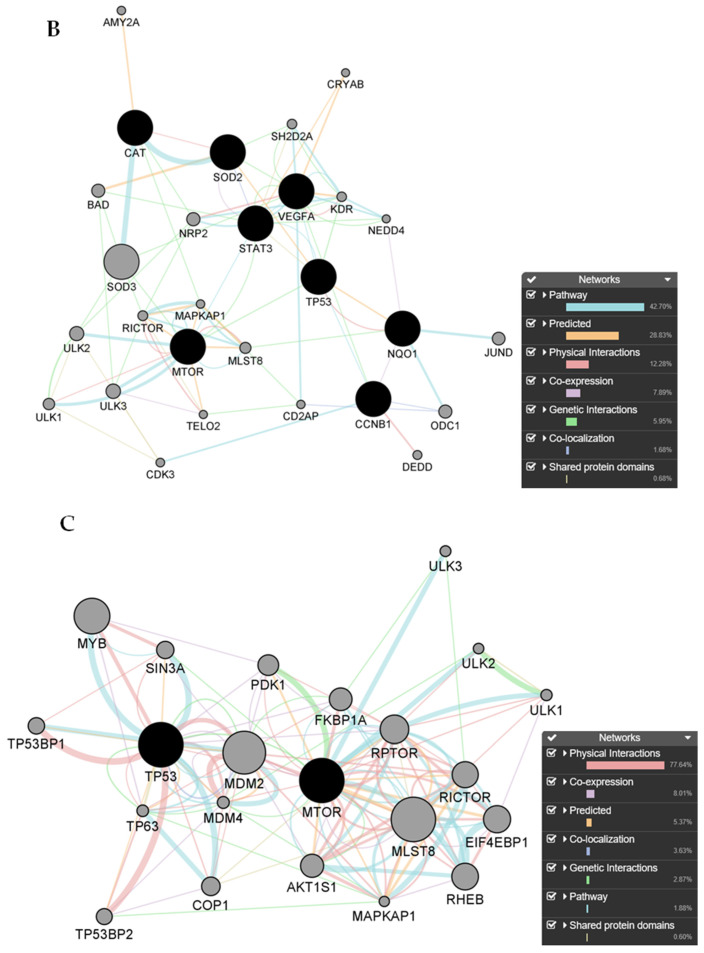
(**A**) Interactions between target genes linked with Pb, Cd, MMC, As, Ni and Cr(VI) exposure and lung cancer, (**B**) NSCLC and (**C**) SCLC, together with 20 related genes (the majority of genes in lung cancer are co-expressed (53.39%), in NSCLC the predominant types of interactions are pathway interactions (42.70%), and in SCLC the predominant types of interactions are physical interactions (77.64%). The original CTD genes (input “seed” genes) are represented by black nodes, while additional genes (interaction partners) predicted by GeneMANIA are represented by smaller gray nodes. The size of the predicted nodes (additional genes predicted by GeneMANIA) reflects computed degree of association (degree to which they are predicted to be related) to the seed nodes, with largest genes representing the most reliable (higher confidence) interactors. The GPX1 gene was not recognized by the GeneMANIA server and was therefore excluded from the network analysis. Source: GeneMANIA.

The GeneMANIA prediction server was subsequently used to analyze the interactions among the common genes associated with Pb, Cd, MMC, As, Ni and Cr(VI) exposure in lung cancer, as well as NSCLC and SCLC. In addition, the analysis identified 20 related genes and characterized the interactions among them. The server categorizes gene interactions into several distinct types: physical interactions, in which proteins encoded by two genes directly interact; co-expression, where genes display similar expression patterns under specific experimental conditions; genetic interactions, in which alterations in one gene affect the function or expression of another gene; co-localization, where genes are expressed within the same tissue or their protein products are localized within the same cellular compartment; pathway-based interactions, in which gene products participate in the same biological pathway or biochemical reaction; and protein domain similarity, referring to proteins that share structurally or functionally related domains, and interactions provided directly by the server [16]. Figure 3 presents the selected key genes along with 20 related genes (predicted interaction partners), illustrating the interactions among them and the contribution of each interaction type. Key genes were selected for lung cancer, as well as NSCLC and SCLC and the results are shown respectively in Figure 3A–C. Gene interaction network visualizations generated using the online GeneMANIA Prediction Server are presented in Supplementary Figures S4–S6.

### 3.3. Toxic Metal(loid)-Gene Interaction

In the subsequent stage of the analysis, the effects of the investigated toxic metal(loid)s on the identified target genes were evaluated using the CTD. The resulting binary chemical–gene interactions are presented in Table 1, Table 2 and Table 3.

The interactions between the examined metal(loid)s and target genes were not uniform. However, several metal(loid)s exhibited concordant effects on protein expression, indicating common patterns of gene regulation. Specifically, Cd, MMC, As, Ni, and Cr(VI) demonstrated concordant effects on VEGFA protein expression in NSCLC, while Pb, Cd, MMC, As, Ni, and Cr(VI) showed concordant effects on HMOX1 protein expression in lung cancer, both leading to increased protein expression. Regarding mRNA expression, Pb, MMC, As, and Ni showed concordant effects on IL6 expression, whereas Pb, Cd, MMC, and Ni exhibited effects on TNF expression in lung cancer, with both resulting in increased mRNA levels. Similarly, Cd, MMC, As, Ni, and Cr(VI) demonstrated concordant effects on VEGFA mRNA expression in NSCLC, leading to increased protein expression. *TP53* was identified as the only gene common to all examined metal(loid)s and all investigated lung cancer categories. Given its well-established role in lung carcinogenesis, this finding suggests that *TP53* may represent a common molecular link between toxic metal(loid) exposure and molecular pathways implicated in lung cancer development. In all investigated lung cancer types, Pb, MMC, and Ni exerted concordant effects on TP53 protein expression, resulting in increased TP53 protein expression. Several interactions exhibited opposing or dual modes of action, reflecting differences in the mechanisms of action of individual metal(loid)s.

**Figure 4:**
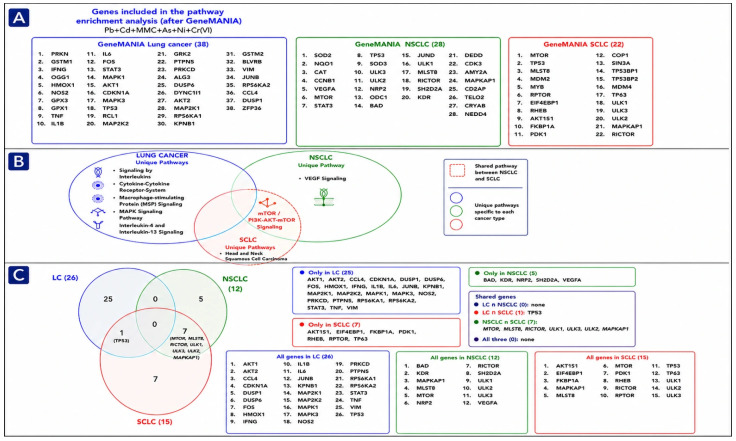
(**A**) Input genes for pathway analysis of lung cancer (LC, umbrella term), NSCLC, and SCLC shared across the investigated toxic metal(loid)s (Pb, Cd, MMC, As, Ni, and Cr(VI)) (GeneMANIA Results). (**B**) Overlapping and unique biological pathways identified from genes shared across the investigated toxic metal(loid)s (Pb + Cd + MMC + As + Ni + Cr(VI)) and LC, NSCLC, and SCLC, together with their 20 related genes predicted by GeneMANIA. Pathways identified by ToppFun were harmonized across the REACTOME, KEGG, WikiPathways (WP), and PID databases and grouped according to biological similarity. The PI3K–AKT–mTOR signaling pathway was the only pathway commonly enriched in both NSCLC and SCLC. (**C**) Overlapping functionally annotated genes associated with lung LC, NSCLC, and SCLC, and shared across the investigated toxic metal(loid)s (Pb, Cd, MMC, As, Ni, and Cr(VI)). Genes were identified using the CTD and expanded with 20 related genes predicted by GeneMANIA. Functional enrichment analysis was performed using ToppFun, and the genes belonging to the five most significantly enriched pathways for each cancer category are shown. Abbreviations: LC, lung cancer; NSCLC, non-small-cell lung cancer; SCLC, small-cell lung cancer; CTD, Comparative Toxicogenomics Database.

Subsequently, the ToppFun pathway enrichment analysis was employed to examine the associations between signaling pathways implicated in the development of the selected diseases and the target genes, together with the 20 related genes identified for lung cancer (based on GeneMANIA), as well as NSCLC and SCLC. The lists of top five significant pathways were delineated based on statistical significance separately for each cancer category and compared to each other using Venn diagram. This resulted into delineation of common and unique enriched pathways. Additionally, the genes statistically associated with each of these five pathways lists were compared using Venn diagram to highlight pathway-focused genetic similarity and differences on cancer category level. This resulted into detection of unique and shared genes in the context of the cancer category-related pathways. The corresponding results are presented in Figure 4 and Supplementary Table S2 for lung cancer, Supplementary Table S3 for NSCLC, and Supplementary Table S4 for SCLC.

## 4. Discussion

Environmental exposures typically occur as complex mixtures of chemical pollutants, including toxic metal(loid)s, rather than as single-substance exposures. Consequently, the biological effects of combined exposures may differ from those of individual toxicants because interactions among mixture components may result in additive, synergistic, or antagonistic effects [17,18,19,20,21]. Despite extensive research on the carcinogenic properties of individual metal(loid)s, including Pb, Cd, MMC, As, Ni, and Cr(VI), with substantial evidence supporting their involvement in lung cancer development, considerably less is known about their combined effects, particularly the molecular mechanisms and biological pathways underlying mixture-associated toxicity [21,22,23,24,25]. Addressing this challenge requires mechanistic approaches capable of identifying genes, molecular networks, and signaling pathways affected by complex exposures [26,27]. In this regard, in silico toxicogenomic approaches provide a powerful framework for integrating existing biological data, predicting toxicant–gene interactions, and identifying molecular pathways potentially involved in disease development, thereby generating mechanistically informed hypotheses for further investigation [21,28,29,30]. Accordingly, the present study investigated common genes, predicted concordant and divergent patterns of gene regulation, and signaling pathways commonly associated with the investigated toxic metal(loid)s (Pb, Cd, MMC, As, Ni, and Cr(VI)) and lung carcinogenesis, while also examining potential differences in the molecular mechanisms underlying SCLC and NSCLC. However, the present study was not designed to directly evaluate interactions among mixture components or to quantitatively assess additive, synergistic, or antagonistic effects. Rather, it provides a hypothesis-generating framework for identifying common toxicogenomic associations and molecular pathways shared across the investigated toxic metal(loid)s that may be relevant to lung carcinogenesis.

### 4.1. Interactions

The interactions between genes associated with Pb, Cd, MMC, As, Ni, and Cr(VI) were not uniform across the analyzed lung cancer types. While some metal(loid)s exhibited concordant effects on target genes, indicating common patterns of regulation, others showed divergent or even opposing effects, reflecting the complexity of toxicogenomic responses associated with multiple toxic metal(loid)s [20,21,25]. Further experimental studies employing dedicated mixture models and quantitative analyses are required to determine whether these concordant and opposing effects result in additive, synergistic, or antagonistic biological responses. Therefore, genes demonstrating consistent responses across multiple metal(loid)s may be of particular interest as potential biomarkers and molecular mediators of metal(loid)-associated lung carcinogenesis. The identification of commonly affected genes is consistent with systems toxicology approaches, which emphasize the importance of shared molecular networks and pathways in understanding the biological consequences of complex environmental exposures [26,30,31].

#### 4.1.1. Concordant Interactions

The following sections first describe the observations obtained from the present computational analysis and subsequently discuss published experimental and clinical evidence to place these findings into biological context.

##### HMOX1-Lung Cancer (Umbrella Term)

The present in silico analysis predicted concordant effects of Pb, Cd, MMC, As, Ni, and Cr(VI) on HMOX1 protein expression in lung cancer, resulting in increased HMOX1 levels. The biological relevance of this prediction was subsequently evaluated in the context of published experimental studies investigating the role of HMOX1 in lung carcinogenesis.

*HMOX1* appears to have a complex and context-dependent role in lung carcinogenesis, acting as part of an antioxidant defense response but also supporting survival of stressed or transformed cells. Kikuchi et al. (2005) reported that the long (GT)n repeat polymorphism in the *HMOX1* promoter, associated with reduced *HMOX1* inducibility, was linked to increased susceptibility to lung adenocarcinoma, particularly among male smokers, suggesting that insufficient *HMOX1* induction may weaken protection against cigarette smoke-related oxidative injury during tumor initiation [32]. In contrast, Li et al. (2008) showed that HMOX1 was significantly increased in lung tumors from smokers and functionally contributed to NNK-induced lung cancer cell proliferation and survival through ERK1/2- and NF-κB-dependent regulation of p21, BCL2, c-IAP2, and BAD signaling [33]. Zhang et al. (2019) further demonstrated that HMOX1 and Nrf2 were elevated in human lung tumors and that HMOX1 inhibition disrupted redox homeostasis and mitochondrial function, leading to oxidative stress accumulation and apoptosis in sensitive lung cancer cells [34]. Consistent with a role for *HMOX1* in metal-induced lung carcinogenesis, chronic Cd exposure was shown to drive malignant transformation of human lung epithelial cells and was accompanied by sustained activation of oxidative stress-response genes, including *HMOX1* and *HIF1A*, alongside the development of multiple cancer-associated characteristics such as increased invasion, hyperproliferation, anchorage-independent growth, and epithelial-to-mesenchymal transition [35]. In a well-differentiated human air–liquid-interface airway tissue model, Cd induced a marked increase in HMOX1 expression that persisted even after a 10-day recovery period, indicating sustained oxidative stress. This persistent oxidative response was accompanied by enhanced inflammatory signaling, matrix metalloproteinase release, tissue remodeling, and squamous differentiation, changes considered relevant to the development of respiratory carcinogenesis [36]. Similarly, low-dose Cr(VI) exposure induced marked HMOX1 overexpression in BEAS2B bronchial epithelial cells, while only limited *HMOX1* induction was observed in A549 lung adenocarcinoma cells [37]. This differential response was accompanied by transient glutathione depletion in BEAS2B cells and was interpreted as evidence of oxidative stress generated during intracellular Cr(VI) reduction, supporting a role for HMOX1 as a key component of cellular defense mechanisms activated by Cr exposure in the respiratory tract [37]. Hard-metal tungsten carbide and metallic cobalt particles also strongly induced HMOX1 and activated a signaling cascade involving p38 MAPK, HIF-1α, and p53, which the authors suggested may contribute to the carcinogenic activity of these occupational particles [38]. Taken together, these findings suggest that *HMOX1* may represent an important mechanistic link between metal-induced oxidative stress and lung carcinogenesis [35,37,38].

Collectively, these published findings support the biological plausibility of the *HMOX1* upregulation predicted in the present computational analysis. Accordingly, the concordant *HMOX1* upregulation predicted following exposure to Pb, Cd, As, Ni, and Cr(VI) may be relevant to oxidative stress adaptation during lung carcinogenesis [33,34,35].

##### IL6—Lung Cancer (Umbrella Term)

Regarding mRNA expression, Pb, MMC, As, and Ni were predicted to exert concordant effects on IL6 expression in lung cancer, resulting in increased transcript levels. To place this prediction into biological context, evidence from previous experimental studies investigating *IL6* in lung carcinogenesis was reviewed [39].

Elevated IL-6 signaling has been associated with lung cancer progression, therapeutic resistance, and poor clinical outcomes, and *IL-6* contributes to the tumor-promoting microenvironment through activation of downstream pathways such as STAT3 [39]. Consistent with this concept, Leng et al. (2016) reported that functional *IL6* promoter variants associated with increased susceptibility to lung squamous cell cancer were linked to higher basal IL6 expression, enhanced promoter activity, and greater IL-6 secretion following carcinogen-induced oxidative stress, supporting the biological relevance of elevated IL-6 signaling in lung carcinogenesis [40]. Furthermore, Ni exposure has been shown to increase IL-6 production in NSCLC cells through activation of the TLR4/MyD88/NF-κB signaling pathway, concomitantly enhancing migratory and invasive behavior [41]. Cadmium has likewise been reported to modulate NF-κB-dependent cytokine responses in pulmonary cells, demonstrating that inflammatory pathways regulating IL6 expression are responsive to metal exposure [42].

Overall, these published studies support the biological plausibility of the *IL6* expression pattern predicted in the present computational analysis. Accordingly, the predicted concordant upregulation of *IL6* following exposure to Pb, MMC, As, and Ni may contribute to the establishment of a persistent pro-inflammatory microenvironment that favors lung carcinogenesis and tumor progression.

##### VEGFA—NSCLC

In NSCLC, Cd, MMC, As, Ni, and Cr(VI) were predicted to exert concordant effects on both VEGFA protein and mRNA expression, resulting in increased expression levels. To interpret these computational findings, published evidence regarding the role of *VEGFA* in NSCLC was examined.

As the principal member of the VEGF family, VEGFA regulates the formation and remodeling of the tumor vasculature through activation of VEGF receptors expressed on endothelial cells [43]. In addition to stimulating neovascularization, VEGFA increases vascular permeability and contributes to the establishment of a microenvironment that supports tumor expansion and dissemination [44,45]. VEGFA expression is frequently induced under hypoxic conditions and has been linked to multiple aspects of cancer progression, including angiogenesis, invasion, and metastatic spread [43,44]. Consistent with its biological importance, therapeutic strategies targeting the VEGFA/VEGFR signaling axis have become an established component of treatment for selected patients with advanced NSCLC [43].

Several studies provide further evidence for the involvement of *VEGFA* in lung cancer biology. In the H125 NSCLC xenograft model, Christensen et al. (2005) demonstrated that inhibition of ErbB signaling significantly reduced both *VEGF* transcript expression and circulating VEGF levels, whereas plasma VEGF concentrations were positively associated with tumor burden [46]. These observations suggest that *VEGF* functions downstream of oncogenic growth-factor signaling pathways and may reflect disease activity. Likewise, Gieseler et al. (2007) detected elevated VEGF levels in malignant effusions from patients with advanced cancers, including lung cancer, and proposed that *VEGF* contributes to the maintenance of a microenvironment favorable for tumor cell survival, invasion, and metastatic progression [47]. Evidence linking environmental carcinogens to *VEGFA* regulation was provided by Wu et al. (2019) [48], who showed that Ni exposure enhanced VEGF-A secretion in lung cancer cells through a mechanism involving TGF-β signaling and integrin β3 upregulation. Notably, suppression of integrin β3 markedly attenuated this response, indicating a direct mechanistic connection between Ni exposure and *VEGFA*-mediated pro-angiogenic signaling [48]. In addition, transcriptomic analysis by Wang et al. (2022) identified significantly higher *VEGFA* expression in lung adenocarcinomas arising in patients with a previous history of breast cancer compared with single primary lung adenocarcinomas, further supporting a role for *VEGFA* in lung tumor development [43].

Collectively, these published studies support the biological plausibility of the *VEGFA*-related predictions generated in the present computational analysis. Accordingly, the predicted concordant upregulation of *VEGFA* following exposure to Cd, MMC, As, Ni, and Cr(VI) may contribute to NSCLC pathogenesis by enhancing angiogenic signaling and promoting a tumor-supportive microenvironment.

##### TP53—Recognized in SCLC and Common Gene for SCLC, NSCLC and Lung Cancer (Umbrella Term)

In the case of SCLC, this in silico analysis indicated that Pb, MMC, and Ni increase TP53 protein expression, whereas Cd, As and Cr(VI) exhibited dual effects. These results suggest that the predicted modulation of *TP53* expression commonly associated with the investigated toxic metal(loid)s may be associated with alterations in apoptotic processes relevant to SCLC biology.

Integrative genomic analyses identified *TP53* as one of the principal driver genes in SCLC, with *TP53* and *RB1* representing the only genes that remained highly significant after rigorous filtering for biologically relevant driver alterations [49]. Moreover, at least one *TP53* allele was altered by mutation, deletion, loss of heterozygosity, or genomic rearrangement in all examined SCLC cases, indicating that *TP53* inactivation is a consistent feature of this malignancy. The same study further demonstrated that *TP53* alterations arise as early clonal events during tumor evolution and concluded that *TP53* inactivation is an early and critical event in the development of human SCLC [49]. Consistent with these findings, Rudin et al. (2012) also reported a high prevalence of inactivating *TP53* mutations and identified *TP53* among the significantly mutated genes defining the genomic landscape of SCLC [50]. Collectively, these studies support a central role for *TP53* disruption in SCLC, although they evaluated genomic alterations rather than TP53 protein expression, limiting direct comparison with the present findings.

The present study indicated that in NSCLC, Pb, MMC, and Ni were predicted to increase TP53 protein expression, whereas Cd, As, and Cr(VI) exhibited dual effects. In addition, *TP53* mRNA expression was predicted to decrease following exposure to Cd, MMC, and Ni. To interpret these observations, previously published studies investigating *TP53* alterations in lung cancer were reviewed. Although Andujar et al. (2013) found no significant difference in overall *TP53* mutation frequency between asbestos-exposed and unexposed NSCLC patients, asbestos exposure was associated with a higher proportion of *TP53* G:C to T:A transversions and increased frequency of *TP53* intron 7 polymorphisms, suggesting that asbestos may influence the *TP53* mutational spectrum and contribute to susceptibility to asbestos-related thoracic malignancies [51]. Beyond its established role in NSCLC pathogenesis, TP53 alterations may also contribute to tumor progression. Amelio et al. (2018) demonstrated that mutant p53 proteins acquire gain-of-function properties and cooperate with hypoxia-inducible factor-1 (HIF-1) to regulate extracellular matrix components and other protumorigenic genes [52]. Expression of this mutant p53/HIF-1-associated transcriptional program was associated with hypoxic tumors and poor prognosis in NSCLC patients, indicating that *TP53* alterations may promote disease progression through the acquisition of novel oncogenic functions [52].

While aforementioned studies highlight the contribution of *TP53* alterations to NSCLC progression, other evidence indicates that environmental metals can directly influence p53 signaling. The available evidence suggests that Pb and methylmercury chloride (MeHgCl) can modulate p53 signaling in lung cells, primarily through oxidative stress-induced apoptotic mechanisms. Pb exposure was shown to increase p53 expression in human lung cancer A549 cells, while MeHgCl induced both p53 expression and activation of p53-associated apoptotic mediators, including Bax, cytochrome c, and caspase-3 in a human lung invasive cancer cell line (Cl1-0), supporting a central role of the p53 pathway in metal-induced pulmonary toxicity and cell death [53,54,55].

Recent genomic studies further support a link between heavy metal exposure and TP53 alterations in NSCLC. Mu et al. (2023) reported a significant positive correlation between *TP53* mutations and serum Pb levels, while Yao et al. (2024) observed a significant positive correlation between *TP53* mutations and Cd levels in patients with advanced NSCLC, suggesting that heavy metal accumulation may be associated with *TP53*-related molecular alterations in lung cancer [56,57].

Considering lung cancer as an umbrella disease term, the present computational analysis predicted that Pb, MMC, and Ni increase TP53 protein expression, whereas Cd, MMC, and Ni decrease *TP53* mRNA expression. Collectively, the available literature provides biological context for these *TP53*-related predictions and highlights the complex and subtype-specific role of *TP53* in lung tumorigenesis. Accordingly, the predicted modulation of *TP53* expression by the investigated toxic metal(loid)s may influence p53-related signaling pathways involved in lung carcinogenesis. However, the biological significance of these findings should be interpreted cautiously given the diverse genetic and functional alterations affecting *TP53* activity and requires further experimental validation.

#### 4.1.2. Interactions Among Common Genes Associated with Pb, Cd, MMC, As, Ni and Cr(VI) Exposure and Different Lung Cancer Types

Interestingly, the predominant patterns of gene–gene interactions differed among the predicted interaction networks generated for the investigated lung cancer categories. Physical interactions represented the dominant interaction category in SCLC (77.64%), whereas pathway-based interactions predominated in NSCLC (42.70%). In contrast, genes associated with lung cancer were primarily linked through co-expression relationships (53.39%). As these interaction networks were generated using GeneMANIA, which integrates publicly available functional genomics datasets rather than disease-specific experimental observations, these findings should be interpreted as predicted interaction patterns rather than direct evidence of biological differences between lung cancer subtypes. Nevertheless, these predicted patterns may be consistent with the well-established molecular heterogeneity of lung cancer, whereby distinct histological subtypes are characterized by different genomic landscapes and pathway-level regulatory networks [49,50,58]. Small-cell lung cancer is characterized by recurrent alterations in TP53 and RB1, together with frequent changes in transcriptional regulators such as SOX2, which contribute to regulatory programs distinct from those observed in other lung cancer subtypes [49,50]. In contrast, NSCLC exhibits substantial molecular and pathway-level heterogeneity, characterized by diverse oncogenic driver alterations and recurrent activation of signaling networks including EGFR/RTK–RAS–RAF–MAPK and PI3K–AKT–mTOR pathway [58,59]. Moreover, gene co-expression network analyses have been widely used to identify coordinated transcriptional programs and functionally related gene modules in lung cancer, facilitating the identification of biologically relevant pathways, hub genes, and molecular networks associated with disease characteristics and clinical outcomes [60,61].

### 4.2. Signaling Pathways

#### 4.2.1. PI3K–AKT–mTOR Signaling—NSCLC and SCLC

The present computational analysis identified the PI3K–AKT–mTOR signaling pathway among the enriched pathways identified from genes shared across the investigated toxic metal(loid)s (Pb, Cd, MMC, As, Ni, and Cr(VI)) and both NSCLC and SCLC through common genes and an additional set of 20 interacting genes predicted by GeneMANIA. This finding is biologically plausible given the central role of the PI3K–AKT–mTOR axis in regulating cellular proliferation, growth, survival, metabolism, angiogenesis, protein synthesis, and resistance to apoptosis, all of which are critical processes in lung carcinogenesis. Dysregulation of this pathway represents one of the most frequent molecular alterations in NSCLC and may arise through aberrant activation of receptor tyrosine kinases, PI3K alterations, PTEN loss, constitutive AKT signaling, and interactions with key oncogenic drivers such as EGFR, KRAS, and TP53 [62,63,64]. Clinical studies further demonstrate that increased activation of pathway components, including phosphorylated AKT, mTOR, and P70S6K, is associated with advanced disease characteristics and poorer survival in NSCLC, supporting the contribution of PI3K–AKT–mTOR signaling to tumor progression and adverse prognosis [65]. Moreover, activation of Akt/mTOR signaling has been implicated in epithelial–mesenchymal transition, metastatic dissemination, maintenance of cancer stem cell properties, therapeutic resistance, and sustained tumor cell survival, further underscoring its importance in lung cancer biology [66].

The relevance of this pathway extends to SCLC, where accumulating evidence indicates that PI3K–AKT–mTOR signaling contributes to tumor aggressiveness, phenotypic plasticity, and resistance to chemotherapy. Activation of the pathway promotes phenotypic transitions associated with a more chemoresistant state, whereas pharmacological inhibition restores sensitivity to anticancer treatment [67]. Similarly, elevated PI3K–mTOR pathway activity has been linked to reduced responsiveness to PARP inhibition, while combined targeting of PI3K and PARP signaling enhances antitumor efficacy in preclinical SCLC models [68]. Further supporting its functional significance, mTOR has been identified as an essential kinase in a subset of SCLC tumors, and mTOR inhibition suppresses tumor growth while increasing sensitivity to cisplatin–etoposide treatment [69]. In addition, activation of mTOR signaling has been demonstrated in pulmonary neuroendocrine tumors, including SCLC, through the expression of phosphorylated mTOR and its downstream effectors, confirming functional engagement of this pathway in lung neuroendocrine malignancies [70].

Importantly, several of the investigated toxic metal(loid)s have previously been associated with modulation of EGFR-, PI3K-, AKT-, and mTOR-dependent signaling. Experimental evidence indicates that As, Cd, Cr, and Ni can promote carcinogenic processes through activation or dysregulation of these pathways, thereby enhancing cellular proliferation, survival, angiogenesis, and malignant transformation [71,72]. More broadly, toxic metal(loid)s induce oxidative stress, genomic instability, impaired DNA repair, and disturbances in intracellular signaling networks that contribute to carcinogenesis [4]. Considering the established carcinogenic potential of several of the investigated toxic metal(loid)s, the identification of PI3K–AKT–mTOR-related pathways in the present computational analysis supports the biological plausibility that this signaling axis may be relevant to molecular mechanisms associated with lung carcinogenesis. Accordingly, these findings suggest that the PI3K–AKT–mTOR pathway may represent a candidate molecular hub linking toxicogenomic associations shared across the investigated toxic metal(loid)s with biological processes implicated in both NSCLC and SCLC.

#### 4.2.2. Signaling by VEGF—NSCLC

The present computational analysis identified “Signaling by VEGF” among the enriched pathways identified from genes shared across the investigated toxic metal(loid)s (Pb, Cd, MMC, As, Ni, and Cr(VI)) and NSCLC, together with an additional set of 20 related genes predicted by GeneMANIA. This finding is biologically plausible given the established role of VEGF signaling in NSCLC pathogenesis. VEGF and its receptors constitute a key regulatory axis controlling tumor angiogenesis by promoting endothelial-cell proliferation, migration, vascular permeability, and neovascularization through downstream signaling pathways such as PI3K–AKT and MAPK/ERK [73,74]. Dysregulation of the VEGF/VEGFR pathway has been widely implicated in NSCLC progression, metastatic dissemination, and aberrant vascular development [73,74].

The clinical significance of VEGF signaling in lung cancer is supported by evidence showing elevated VEGF expression in patients with NSCLC. Wardhani et al. (2025) demonstrated significantly increased VEGF levels in both serum and bronchial wash samples from NSCLC patients compared with controls, suggesting a close association between VEGF activation and disease development [75]. Similarly, a meta-analysis including 5386 lung cancer patients identified VEGF overexpression as a marker of poor prognosis in both NSCLC and SCLC, indicating that enhanced VEGF signaling is associated with more aggressive disease and reduced survival [76].

Beyond its angiogenic functions, VEGF also contributes to the establishment of a tumor-supportive microenvironment. VEGF-mediated signaling can promote immunosuppressive conditions within NSCLC by influencing interactions among endothelial, stromal, immune, and tumor cells [73]. Consistent with this concept, single-cell transcriptomic analysis by Huang et al. (2025) demonstrated that VEGF-dependent communication between tumor-associated macrophages, fibroblasts, endothelial cells, and tumor cells was linked to resistance to combined anti-angiogenic and immunotherapeutic treatment and to the development of immunosuppressive phenotypes in NSCLC [77].

Experimental evidence further suggests that environmental metal exposure may modulate VEGF-related mechanisms in lung cancer. In human A549 lung adenocarcinoma cells, low-dose Cd exposure increased VEGF expression and secretion and enhanced the angiogenic activity of the tumor microenvironment through stimulation of endothelial-cell proliferation and migration. The same study showed increased HIF-1α expression following Cd exposure, suggesting a potential mechanism underlying VEGF upregulation [78]. Although these findings were obtained for Cd alone, they provide mechanistic support for the hypothesis that metal(loid)-associated perturbation of VEGF signaling may contribute to lung carcinogenesis.

Collectively, the available evidence supports the biological plausibility of the VEGF signaling pathway identified in the present computational analysis and suggests that alteration of VEGF-related signaling may represent one of the molecular mechanisms potentially linking toxicogenomic associations shared across the investigated toxic metal(loid)s with NSCLC development and progression.

#### 4.2.3. Macrophage-Stimulating Protein (MSP) Signaling—Lung Cancer (Umbrella Term)

The present computational analysis identified macrophage-stimulating protein (MSP) signaling among the enriched pathways identified from genes shared across the investigated toxic metal(loid)s (Pb, Cd, MMC, As, Ni, and Cr(VI)) and lung cancer together with an additional set of 20 related genes predicted by GeneMANIA. Although direct evidence linking the investigated toxic metal(loid)s to MSP signaling in lung cancer remains limited, the established biological functions of the MSP/RON axis support the biological plausibility of the MSP signaling pathway identified in the present computational analysis. MSP is the only known ligand of the receptor tyrosine kinase RON (MST1R), and activation of the MSP/RON system triggers several downstream signaling cascades, including PI3K/AKT, MAPK, JNK, and FAK pathways, which regulate cellular proliferation, survival, migration, invasion, angiogenesis, and therapy resistance, all of which are hallmarks of cancer progression [79].

Evidence from lung cancer studies further supports the involvement of MSP signaling in tumor progression. Willett et al. (1998) demonstrated that MSP and RON may function as an autocrine/paracrine signaling system in a subset of NSCLCs, where MSP-induced RON activation resulted in receptor tyrosine phosphorylation and enhanced tumor-cell migration, suggesting a role in promoting tumor invasiveness and disease progression [80]. Similarly, Sato et al. (2013) showed that MSP overexpression significantly increased liver metastasis formation in an experimental SCLC model [81]. Notably, this effect was accompanied by increased macrophage infiltration and tumor-associated microvessel density, indicating that MSP may facilitate metastatic dissemination primarily through modulation of the tumor microenvironment and angiogenesis rather than through direct stimulation of tumor cell proliferation [81]. In addition, MSP has been shown to activate human alveolar macrophages, inducing reactive oxygen species production, NF-κB activation, and the release of inflammatory mediators, including TNF-α and IL-1β, thereby linking MSP signaling to pulmonary inflammatory responses that may contribute to a pro-tumorigenic microenvironment [82].

Considering that several of the downstream pathways associated with MSP/RON signaling, including PI3K/AKT and MAPK, were also identified in the present computational analysis, MSP signaling may represent a potential molecular link between the predicted toxicogenomic associations of the investigated toxic metal(loid)s and biological processes implicated in lung cancer progression, including chronic inflammation, angiogenesis, and metastasis [79,80,81].

#### 4.2.4. MAPK Signaling Pathway—Lung Cancer (Umbrella Term)

The present computational analysis identified the MAPK signaling pathway among the enriched pathways identified from genes shared across the investigated toxic metal(loid)s (Pb, Cd, MMC, As, Ni, and Cr(VI)) and lung cancer, together with an additional set of 20 related genes predicted by GeneMANIA. This finding is biologically plausible given the central role of MAPK signaling in lung cancer pathogenesis. The MAPK cascade, particularly the EGFR–RAS–RAF–MEK–ERK axis, regulates critical cellular processes including proliferation, differentiation, survival, apoptosis, angiogenesis, invasion, and metastasis, and its dysregulation is a frequent event in NSCLC [59]. Aberrant activation of MAPK pathway components has been associated with enhanced tumor growth, resistance to apoptosis, metastatic potential, and therapeutic resistance in NSCLC, highlighting its importance as both a driver of disease progression and a therapeutic target [59].

The available literature further supports a mechanistic connection between toxic metal(loid) exposure and MAPK pathway perturbation. Heavy metals and metalloids are known to induce reactive oxygen species (ROS) generation and oxidative stress, which can activate multiple intracellular signaling pathways, including MAPK cascades, thereby contributing to malignant transformation and carcinogenesis [83]. Arsenic exposure has been shown to activate members of the MAPK family, including ERK1/2, JNK, and p38, resulting in altered cellular proliferation, transformation, and apoptosis depending on exposure conditions [83]. Furthermore, Cd, Ni, and Cr(VI) have been implicated in carcinogenic processes involving oxidative stress, dysregulated signaling, angiogenesis, DNA damage, and malignant transformation [83,84]. Zhao et al. (2022) further emphasized that aberrant signaling pathways, together with epigenetic alterations, constitute important mechanisms underlying Cr-, Ni-, and Cd-induced carcinogenesis [84].

Beyond its direct effects on tumor growth, MAPK signaling also contributes to immune regulation within the lung tumor microenvironment. Stutvoet et al. (2019) demonstrated that MAPK pathway activity is a key regulator of PD-L1 expression in lung adenocarcinoma and that inhibition of MAPK signaling suppresses both growth factor- and cytokine-induced PD-L1 upregulation [85]. These findings indicate that MAPK activation may facilitate immune evasion in addition to promoting tumor cell proliferation and survival [85]. Collectively, the established role of MAPK signaling in lung cancer biology and published evidence that several of the investigated toxic metal(loid)s are associated with oxidative stress and alterations in MAPK-related signaling support the biological plausibility of the MAPK pathway identified in the present computational analysis and suggest that it may represent a candidate molecular mechanism linking toxic metal(loid)-associated toxicogenomic responses with biological processes implicated in lung carcinogenesis [83,84,85].

#### 4.2.5. Signaling by Interleukins, Cytokine Signaling in Immune System, Interleukin 4 and Interleukin 13 Signaling—Lung Cancer (Umbrella Term)

The present computational analysis identified “Signaling by interleukins” as a pathway commonly associated with the investigated toxic metal(loid)s through shared genes and their 20 related genes identified by GeneMANIA. This finding is consistent with growing evidence that dysregulated cytokine signaling is a key driver of lung carcinogenesis, contributing to chronic inflammation, immune modulation, angiogenesis, tumor cell survival, and disease progression. Interleukins are central mediators of communication within the tumor microenvironment, and their aberrant activation has been increasingly recognized as an important feature of lung cancer development [86].

Among the interleukins identified in the present analysis, IL-1β appears to function as an upstream regulator of the inflammatory network. IL-1β promotes activation of multiple signaling pathways, including NF-κB, MAPK, AP-1, and ERK, thereby sustaining a pro-inflammatory microenvironment that favors tumor initiation and progression. In lung cancer, IL-1β has been associated with enhanced angiogenesis, recruitment of immunosuppressive myeloid populations, tumor invasion, and metastatic dissemination. Moreover, IL-1β stimulates the production of downstream inflammatory mediators, particularly IL-6, amplifying and perpetuating tumor-promoting inflammatory responses within the lung microenvironment [87,88].

The enrichment of IL-6 signaling observed in the present study is particularly noteworthy given the extensive evidence supporting its role in lung cancer. Elevated circulating IL-6 levels have consistently been reported in lung cancer patients and have been proposed as a biomarker of disease presence and progression [89]. Mechanistically, IL-6 exerts many of its biological effects through activation of the JAK/STAT3 pathway, promoting cell proliferation, survival, angiogenesis, and immune modulation [90]. Although IL-6 may contribute to maintaining tissue homeostasis during the earliest stages of tumorigenesis, persistent activation of IL-6/STAT3 signaling has been shown to facilitate the growth and progression of established tumors and is associated with adverse clinical outcomes [39]. Notably, Wang et al. (2024) demonstrated that chronic Cr(VI) exposure induces lung adenocarcinoma through activation of the non-canonical NF-κB pathway, resulting in enhanced IL-6/JAK/STAT3 signaling and increased inflammatory and immunosuppressive responses, thereby providing a mechanistic link between metal exposure and lung carcinogenesis [91].

The present findings are further supported by evidence highlighting the importance of the IL-4/IL-13–STAT6 signaling axis in lung cancer progression. Both IL-4 and IL-13 are major regulators of Th2-associated immune responses and alternative macrophage activation and are generally considered protumorigenic cytokines because they promote immune suppression and tumor-supportive inflammation [86,92]. Increased expression of IL-4, IL-13, and STAT6 has been reported in NSCLC tissues, indicating activation of this signaling network during lung tumor development [93]. Furthermore, activation of IL-4/STAT6 signaling promotes the accumulation of M2-polarized macrophages, which contribute to angiogenesis, tissue remodeling, immune evasion, and tumor progression, whereas disruption of this pathway suppresses tumor growth [94]. The relevance of this signaling axis to environmentally induced carcinogenesis is further supported by toxicogenomic analyses identifying IL-4 and IL-13 signaling as major pathways linking toxicant exposure with cancer development and macrophage-mediated tumor promotion.

Collectively, these findings suggest that the enrichment of interleukin signaling commonly associated with the investigated toxic metal(loid)s may reflect coordinated inflammatory and immune-regulatory processes involving IL-1β, IL-6, IL-4, and IL-13. Through their coordinated effects on inflammatory signaling, macrophage polarization, angiogenesis, immune suppression, and tumor cell survival, these cytokines may contribute to the establishment of a persistent pro-inflammatory and tumor-supportive microenvironment that facilitates lung cancer development and progression.

The differences observed between analyses performed at the level of lung cancer overall and those conducted separately for NSCLC and SCLC underscore the biological heterogeneity of lung cancer. These findings suggest that, whenever feasible, subtype-specific approaches should be incorporated into analysis, as they may provide mechanistic insights which may be overlooked when lung cancer is considered as an overall disease entity.

#### 4.2.6. Limitations

This study is limited by its reliance on publicly curated toxicogenomic databases and computational prediction tools, including CTD and GeneMANIA, which depend on the completeness and quality of the available data. The predicted interaction networks generated by GeneMANIA are based on integrated public datasets rather than disease-specific experimental observations and should therefore be interpreted as hypothesis-generating rather than as direct evidence of biological differences between lung cancer subtypes. Additionally, one input gene (GPX1) was not recognized by the GeneMANIA server and was therefore excluded from the generated interaction networks. Furthermore, the study lacks experimental validation, cannot establish causal relationships between toxic metal(loid) exposure and lung carcinogenesis, and does not account for exposure dose, duration, timing, route of exposure, or individual susceptibility. In addition, quantitative exposure data and temporal information are not incorporated into the analyses. Therefore, the identified genes, interaction networks, and signaling pathways should be regarded as hypothesis-generating and require validation through experimental, epidemiological, and mechanistic studies.

## 5. Conclusions

This in silico toxicogenomic study investigated the potential molecular association between the investigated toxic metal(loid)s (Pb, Cd, MMC, As, Ni, and Cr(VI)) and lung carcinogenesis through the integration of curated gene-chemical associations, molecular interaction networks, and pathway enrichment analyses. Two common genes (*MTOR* and *TP53*) were identified for SCLC, eight genes (*CAT*, *CCNB1*, *MTOR*, *NQO1*, *SOD2*, *STAT3*, *TP53*, and *VEGFA*) for NSCLC, and eighteen genes (*AKT1*, *CDKN1A*, *FOS*, *GPX1*, *GPX3*, *GSTM1*, *HMOX1*, *IFNG*, *IL1B*, *IL6*, *MAPK1*, *MAPK3*, *NOS2*, *OGG1*, *PRKN*, *STAT3*, *TNF*, and *TP53*) for lung cancer overall. Gene network analysis revealed that physical interactions predominated in SCLC, pathway interactions in NSCLC, and co-expression relationships in lung cancer. Furthermore, several metal(loid)s were predicted to exert concordant effects on key cancer-related genes, including *TP53* in SCLC, *VEGFA* in NSCLC, and *HMOX1* and *IL6* in lung cancer. Notably, *TP53* was the only gene associated with all investigated metal(loid)s and all examined lung cancer categories. Given the well-established role of *TP53* in lung carcinogenesis, this finding should be interpreted with caution and likely reflects both its central role in lung cancer biology and its involvement in molecular responses to toxic metal(loid) exposure. Nevertheless, its consistent identification across all analyses suggests that *TP53* may represent a common molecular link between toxic metal(loid)-associated toxicogenomic responses and lung carcinogenesis, warranting further investigation in the context of metal(loid)-associated lung carcinogenesis. Pathway enrichment analysis revealed subtype-specific and shared molecular mechanisms, with the PI3K–AKT–mTOR signaling pathway identified in both SCLC and NSCLC, VEGF signaling specifically associated with NSCLC, and MAPK signaling, signaling by interleukins, and macrophage-stimulating protein (MSP) signaling enriched in lung cancer overall. These findings provide mechanistically informed hypotheses regarding molecular mechanisms potentially underlying the association between exposure to the investigated toxic metal(loid)s and lung carcinogenesis and identify *TP53*, *VEGFA*, *HMOX1*, and *IL6*, together with their associated signaling pathways, as candidate molecular markers and potential mechanistic targets for further experimental and epidemiological investigation. The differences observed between the analysis of lung cancer overall and the separate analyses of NSCLC and SCLC suggest that the level of disease classification may influence the toxicogenomic patterns identified. While analysis of lung cancer overall provides a broad overview of shared associations, subtype-specific analyses may reveal additional patterns requiring further investigation. Future studies may therefore benefit from considering individual lung cancer subtypes alongside analyses of lung cancer as a broader disease category.

## Figures and Tables

**Table 1:** Influence of Pb, Cd, MMC, As, Ni and Cr(VI) on key genes linked with lung cancer.

Disease	Lung Cancer						
Gene	Interaction	Lead (Pb)	Cadmium (Cd)	Methylmercury Compounds (MMCs)	Arsenic (As)	Nickel (Ni)	Chromium Hexavalent Ion (Cr(VI))
*AKT1*	mRNA expression				↓		
	protein expression						
*CDKN1A*	mRNA expression		↑↓	↑	↑↓	↑	
	protein expression		↑↓		↓		
*FOS*	mRNA expression		↑↓	↑	↑	↑	
	protein expression		↑				
*GPX1*	mRNA expression	↓	↓	↑↓	↑↓		↓
	protein expression			↑			↓
*GPX3*	mRNA expression			↑	↑		↑
	protein expression		↓				
*GSTM1*	mRNA expression			↓		↓	
	protein expression		↓				
*HMOX1*	mRNA expression	↑↓	↑↓	↑	↑↓	↑↓	
	protein expression	↑	↑	↑	↑	↑	↑
*IFNG*	mRNA expression				↓	↑	
	protein expression			↓			↑
*IL1B*	mRNA expression	↑	↑		↑↓	↑	
	protein expression	↑	↑		↑↓	↑	
*IL6*	mRNA expression	↑	↑↓	↑	↑	↑	↑↓
	protein expression		↑		↑		↑
*MAPK1*	mRNA expression		↑			↑	
	protein expression						
*MAPK3*	mRNA expression						
	protein expression						
*NOS2*	mRNA expression	↑	↑	↓			↑
	protein expression	↑			↓		↑
*OGG1*	mRNA expression		↓		↑↓		↓
	protein expression		↓		↑↓		
*PRKN*	mRNA expression	↓					
	protein expression		↑				↑
*STAT3*	mRNA expression				↑↓	↑	
	protein expression						↑
*TNF*	mRNA expression	↑	↑	↑	↑↓	↑	
	protein expression	↑	↑		↑↓	↑	
*TP53*	mRNA expression	↑↓	↓	↓	↑↓	↓	↑
	protein expression	↑	↑↓	↑	↑↓	↑	↑↓

↑—increases; ↓—decreases; ↑↓—increases and decreases.

**Table 2:** Influence of Pb, Cd, MMC, As, Ni and Cr(VI) on key genes linked with NSCLC.

Disease	Non-Small-Cell Lung Cancer						
Gene	Interaction	Lead (Pb)	Cadmium (Cd)	Methylmercury Compounds (MMCs)	Arsenic (As)	Nickel (Ni)	Chromium Hexavalent Ion (Cr(VI))
*CAT*	mRNA expression	↓	↑↓				↓
	protein expression	↓					↑↓
*CCNB1*	mRNA expression		↓			↑	
	protein expression		↑		↑		↑
*MTOR*	mRNA expression				↓		
	protein expression		↑		↑		↑
*NQO1*	mRNA expression	↑↓	↑↓	↑	↑↓	↑	↓
	protein expression		↑↓	↑	↑		
*SOD2*	mRNA expression	↓	↑↓	↑↓	↑↓	↑	
	protein expression			↑	↑		↑
*STAT3*	mRNA expression				↑↓	↑	
	protein expression						↑
*TP53*	mRNA expression	↑↓	↓	↓	↑↓	↓	↑
	protein expression	↑	↑↓	↑	↑↓	↑	↑↓
*VEGFA*	mRNA expression		↑	↑	↑	↑	↓
	protein expression		↑	↑	↑	↑	↑

↑—increases; ↓—decreases; ↑↓—increases and decreases.

**Table 3:** Influence of Pb, Cd, MMC, As, Ni and Cr(VI) on key genes linked with SCLC.

Disease	Small-Cell Lung Cancer						
Gene	Interaction	Lead (Pb)	Cadmium (Cd)	Methylmercury Compounds (MMCs)	Arsenic (As)	Nickel (Ni)	Chromium Hexavalent Ion (Cr(VI))
*MTOR*	mRNA expression						
	protein expression		↑		↑		↑
*TP53*	mRNA expression	↑↓	↓		↑↓	↓	↑
	protein expression	↑	↑↓	↑	↑↓	↑	↑↓

↑—increases; ↓—decreases; ↑↓—increases and decreases.

## Data Availability

The authors confirm that the data supporting the findings of this study are available within the article, the source data, and its Supplementary Materials.

## Supplementary Materials

The following supporting information can be downloaded at https://www.mdpi.com/article/10.3390/cancers18152395/s1, Table S1: Genes associated with lung cancer, NSCLC and SCLC, identified for each investigated toxic metal(loid) (Pb, Cd, MMC, As, Ni, and Cr(VI)) and the corresponding shared genes (based on MyVenn tool). Figure S1: Common target genes associated with Pb, Cd, MMC, As, Ni and Cr(VI) exposure and lung cancer. Figure S2: Common target genes associated with Pb, Cd, MMC, As, Ni and Cr(VI) exposure and NSCLC. Figure S3: Common target genes associated with Pb, Cd, MMC, As, Ni and Cr(VI) exposure and SCLC. Figure S4: Interactions between target genes linked with Pb, Cd, MMC, As, Ni and Cr (VI) exposure and lung cancer, together with 20 related genes. The target genes common to the investigated toxic metal(loid) are represented as striped nodes (circles with diagonal line) while circles without diagonal line indicate related genes. The size of the predicted nodes (additional genes predicted by GeneMANIA) reflects computed degree of association (degree to which they are predicted to be related) to the seed nodes, with largest genes representing the most reliable (higher confidence) interactors. Figure S5: Interactions between target genes linked with Pb, Cd, MMC, As, Ni and Cr (VI) exposure and NSCLC, together with 20 related genes. The target genes common to the investigated toxic metal(loid) are represented as striped nodes (circles with diagonal line) while circles without diagonal line indicate related genes. The size of the predicted nodes (additional genes predicted by GeneMANIA) reflects computed degree of association (degree to which they are predicted to be related) to the seed nodes, with largest genes representing the most reliable (higher confidence) interactors. Figure S6: Interactions between target genes linked with Pb, Cd, MMC, As, Ni and Cr (VI) exposure and SCLC, together with 20 related genes. The target genes common to the investigated toxic metal(loid) are represented as striped nodes (circles with diagonal line) while circles without diagonal line indicate related genes. The size of the predicted nodes (additional genes predicted by GeneMANIA) reflects computed degree of association (degree to which they are predicted to be related) to the seed nodes, with largest genes representing the most reliable (higher confidence) interactors. Table S2: Enriched signaling pathways identified using genes shared across the investigated toxic metal(loid)s (Pb, Cd, MMC, As, Ni, and Cr(VI)) and lung cancer, together with their 20 related genes identified by GeneMANIA (based on CTD, GeneMANIA and ToppFun tool). Table S3: Enriched signaling pathways identified using genes shared across the investigated toxic metal(loid)s (Pb, Cd, MMC, As, Ni, and Cr(VI)) and NSCLC, together with their 20 related genes identified by GeneMANIA (based on CTD, GeneMANIA and ToppFun tool). Table S4: Enriched signaling pathways identified using genes shared across the investigated toxic metal(loid)s (Pb, Cd, MMC, As, Ni, and Cr(VI)) and SCLC, together with their 20 related genes identified by GeneMANIA (based on CTD, GeneMANIA and ToppFun tool).

## Abbreviations

The following abbreviations are used in this manuscript:

A549	Human lung adenocarcinoma cell line
AKT	Protein kinase B
AKT1	AKT serine/threonine kinase 1
As	Arsenic
ATSDR	Agency for Toxic Substances and Disease Registry
BAD	BCL2-associated agonist of cell death
BAX	BCL2-associated X protein
BCL2	B-cell lymphoma 2
BEAS-2B	Human bronchial epithelial cell line
c-IAP2	Cellular inhibitor of apoptosis protein 2
CAT	Catalase
CCNB1	Cyclin B1
Cd	Cadmium
Cl1-0	Human lung invasive cancer cell line
Cr(VI)	Hexavalent chromium
CTD	Comparative Toxicogenomics Database
DNA	Deoxyribonucleic acid
EGFR	Epidermal growth factor receptor
ERK1/2	Extracellular signal-regulated kinase 1/2
ErbB	ErbB family of receptor tyrosine kinases
FAK	Focal adhesion kinase
FOS	Fos proto-oncogene
G:C	Guanine:cytosine
T:A	Thymine:adenine
GPX1	Glutathione peroxidase 1
GPX3	Glutathione peroxidase 3
GSTM1	Glutathione S-transferase Mu 1
H125	Human lung cancer cell line
HIF-1	Hypoxia-inducible factor 1
HIF-1α	Hypoxia-inducible factor 1 alpha
HIF1A	Hypoxia-inducible factor 1 subunit alpha
HMOX1	Heme oxygenase 1
IARC	International Agency for Research on Cancer
IFNG	Interferon gamma
IL1B	Interleukin 1 beta
IL4	Interleukin 4
IL6	Interleukin 6
IL13	Interleukin 13
Integrin β3	Integrin beta 3
JAK	Janus kinase
JNK	c-Jun N-terminal kinase
KRAS	KRAS proto-oncogene
MAPK	Mitogen-activated protein kinase
MAPK1	Mitogen-activated protein kinase 1
MAPK3	Mitogen-activated protein kinase 3
MeHgCl	Methylmercury chloride
MMC	Methylmercury compounds
mRNA	Messenger ribonucleic acid
mTOR	Mechanistic target of rapamycin
MSP	Macrophage-stimulating protein
MTOR	Mechanistic target of rapamycin kinase
MyD88	Myeloid differentiation primary response protein 88
NF-κB	Nuclear factor kappa B
Ni	Nickel
NNK	4-(Methylnitrosamino)-1-(3-pyridyl)-1-butanone
NOS2	Nitric oxide synthase 2
NQO1	NAD(P)H quinone dehydrogenase 1
NSCLC	Non-small-cell lung cancer
OGG1	8-Oxoguanine DNA glycosylase 1
P70S6K	70 kDa ribosomal protein S6 kinase
PARP	Poly(ADP-ribose) polymerase
Pb	Lead
PD-L1	Programmed death-ligand 1
PI3K	Phosphoinositide 3-kinase
PRKN	Parkin RBR E3 ubiquitin protein ligase
PTEN	Phosphatase and tensin homolog
p38 MAPK	p38 mitogen-activated protein kinase
p53	Tumor protein p53
p21	Cyclin-dependent kinase inhibitor 1A
RB1	RB transcriptional corepressor 1
RAS	Rat sarcoma viral oncogene homolog
RAF	Rapidly accelerated fibrosarcoma kinase
RON	Recepteur d’origine nantais (macrophage-stimulating 1 protein receptor)
ROS	Reactive oxygen species
RTK	Receptor tyrosine kinase
SCLC	Small-cell lung cancer
SOD2	Superoxide dismutase 2
SOX2	SRY-box transcription factor 2
STAT3	Signal transducer and activator of transcription 3
STAT6	Signal transducer and activator of transcription 6
TGF-β	Transforming growth factor beta
TLR4	Toll-like receptor 4
TNF	Tumor necrosis factor
TP53	Tumor protein p53
VEGF	Vascular endothelial growth factor
VEGFA	Vascular endothelial growth factor A
VEGFR	Vascular endothelial growth factor receptor
WHO	World Health Organization
(GT)n	Guanine–thymine dinucleotide repeat
