# Supplementary material for: Toxicogenomic Characterization of Common Genes and Signaling Pathways Associated with Multiple Toxic Metal(loid)s in Lung Cancer: A Comparative Analysis of Small-Cell and Non-Small-Cell Lung Cancer
Source: Cancers (Basel). 2026 Jul 25;18(15):2395. doi: 10.3390/cancers18152395 (PMC13465796; doi:10.3390/cancers18152395)
Supplement: Supplementary file 1 [file cancers-18-02395-s001.zip › cancers-4439574-supplementary.pdf]

Table S1. Genes associated with lung cancer, NSCLC and SCLC, identified for each investigated toxic metal(loid) (Pb, Cd, MMC, As, Ni, and Cr(VI)) and the corresponding shared genes (based on MyVenn tool).

	Total number of genes	**Lung Cancer**	**Non-Small Cell Lung Cancer**	**Small Cell Lung Cancer**
Lead (Pb)	**3629**	*A2M, ACE, ACSM1, ACTB, AKT1, ANK3, ANXA2, APOA1, APOE, AZGP1, BCL2L1, BECN1, CA12, CASP8, CCND1, CDKN1A, CDKN1C, CHD4, CHEK2, CPE, CRP, CTNNB1, CYP1A2, DAPK1, DNMT3A, EGFR, EGR1, EHMT2, EPHX1, ERBB2, FAS, FOS, FOSL2, FOXM1, FOXP3, FUBP1, GATA6, GC, GCLC, GJA1, GPX1, GPX3, GSTM1, GSTP1, GSTT1, HES1, HMOX1, HRAS, IDS, IFNG, IL10, IL1B, IL6, JUN, KRAS, MAP4K4, MAPK1, MAPK3, MET, MIR10A, MIR146A, MIR21, MIR222, MIR34B, MIR34C, MIR494, NFE2L2, NOS2, NOTCH2, OGG1, PDCD1, PDCD4, PON1, PRDX1, PRKN, PTEN, RAMP2, ROBO1, SELENOP, SERPINA1, SERPING1, SLC3A2, SMARCC1, SND1, SPP1, STAT3, STIM1, STN1, TERT, TFRC, TGFB1, TGFBR2, TLR4, TNF, TP53, TSC2, TSHR, TTR, WT1*	*ABCB1, ADAMTS1, AKR1C1, AVEN, CA9, CASP8, CAT, CCNB1, CD74, CD9, CERT1, CHGA, CXCL8, DAPK1, E2F2, E2F7, EGFR, ENO2, FOXM1, FOXO3, GCLC, GRIK2, GSTP1, HES1, IL10, ITGB4, KDM1A, KEAP1, KRAS, LGALS1, MACIR, MET, MTOR, NFE2L2, NNAT, NQO1, OSMR, PGAM1, PRDX1, PTHLH, RB1, RHEB, RRM1, SDC4, SOD2, STAT3, SYP, TERT, TP53, VEGFA*	*MTOR, RB1, TP53, COL4A2, EGFR, ELAVL2, EP300, ID2, INSM1, KHSRP, KIF21A, PLSCR4, PTEN, RUNX1T1, SATB2, ZDBF2*
Cadmium (Cd)	**4889**		*ABCB1, AKR1C1,*	*BIRC5, MTOR,*

		A2M, ACE, ADA, AKT1, ALX4, ANXA2, APC, APOA1, APOC3, APOE, AR, ATG101, ATOX1, AVPI1, AZGP1, BAP1, BCL2L1, BECN1, BHLHE41, BIRC5, BRAF, BRCA2, CA12, CALML3, CASP8, CAV1, CBR2, CCN1, CCN2, CCND1, CD274, CDH13, CDKN1A, CDKN1B, CDKN1C, CDKN2A, CEACAM1, CES1, CES1F, CHEK2, CHRNA7, CLCA2, COL6A1, COX17, CPE, CRP, CTNNB1, CXCL14, CYP1A2, CYP1B1, CYP24A1, CYP2E1, DAB2IP, DAPK1, DDR1, DNMT3A, DOK1, EEF2, EFEMP1, EFNB2, EGFR, EGR1, EHMT2, EPHX1, ERBB2, ERBB3, ERGIC3, ESR1, FAS, FASLG, FEN1, FGFR1, FGFR2, FOS, FOSB, FOSL2, FOXM1, FUBP1, GAST, GC, GCLC, GJA1, GJB1, GPX1, GPX3, GRB7, GSTM1, GSTP1, GSTT1, HES1, HEY1, HILPDA, HMOX1, HRAS, HTRA1, ID3, IDS, IFNG, IKBKG, IL10, IL1B, IL1R2, IL2, IL6, JAG1, JUN, JUNB, JUND, KDR, KRAS, MAP2K7, MAP3K8, MAPK1, MAPK14, MAPK3, MCL1, MET, MIR146A, MIR155, MIR21,	*APC, AREG, ATF3, AVEN, AXL, BIRC5, CASP8, CAT, CCNB1, CD74, CD9, CDH13, CDKN2A, CHFR, CHGA, COL7A1, CORO1C, CSF3, CST6, CXCL8, CYLD, CYP2E1, DAPK1, DEUP1, DUSP3, E2F1, E2F5, E2F7, E2F8, EGFR, ENO1, ENO2, ERGIC3, FASLG, FGFR1, FOXA1, FOXM1, FOXO3, GCLC, GIPR, GSTM2, GSTP1, HES1, HIF1A, IL10, ITGB1, ITGB4, KDM1A, KEAP1, KIF5B, KIT, KRAS, LAMC2, LGALS1, LRRC59, MDM2, MET, MIR29A, MIR346, MMP1, MMP11, MMP9, MT3, MTOR, MYC, NCOA3, NDRG1, NEK2, NFE2L2, NNAT, NOTCH3, NQO1, OSMR, OXTR, PGAM1, PHGDH, PHLDA2, PLBD1, PPIA, PRDX1, PRR13, PSAT1, PTHLH, PXN, PYCARD, RAF1, RASSF1, RB1, RBM7, RHEB, ROS1, RRAD, RRM1, SDC4, SELENOS, SHMT2, SOCS2, SOD2, STAT3, SYP, TERT, TGFA, TMEM135, TP53, UCHL1, VEGFA, WEE1, XPC*	*NPPA, PYCARD, RB1, TP53, UGT1A1, ADCY1, ASCL1, BCLAF1, CKB, COL22A1, COL4A2, CREBBP, CSF3, EGFR, EP300, FGFR1, GRM8, ID2, KIF21A, KIT, KMT2A, MYCL, NDRG1, PLSCR4, PTEN, RUNX1T1, SATB2, SLIT2, ZDBF2*

		MIR222, MIR224, MIR302D, MIR30A, MIR34B, MIR410, MIR98, MMP1, MMP10, MPO, MPP1, MTHFR, MUC12, MYC, NFE2L2, NOS2, NOTCH2, NOTCH3, NPPA, NRG1, OGG1, PCNA, PDLIM4, PGGT1B, PIK3CA, PON1, PPBP, PPP2R1B, PRDX1, PRDX6, PRKN, PTEN, PTGIS, PTMA, PYCARD, RAF1, RARB, RASSF1, RCHY1, RTEL1, SELENBP1, SELENOP, SERPINA1, SERPING1, SFTPB, SIDT2, SLC3A2, SMC2, SOX9, SPP1, STAT3, STIM1, STK11, TEP1, TERT, TFRC, TGFB1, TGFBR2, TLR4, TNF, TP53, TP63, TRP53, TTR, TYMS, USP18, VHL, WNT5A, WT1, XPC		
Methylmercury compounds (MMC)	**9644**	*ABCB1, APC, CAT, CCNB1, CDKN2A, CYP2E1, DUSP3, EGFR, ERGIC3, GCLC, GRIK2, GSTP1, HIF1A, KEAP1, MDM2, MET, MMP9, MTOR, NFE2L2, NQO1, RAF1, SOD2, STAT3, SYP, TERT, TP53, UCHL1, VEGFA, CKB, ID2, BCL10, BCL2, C9, CCL3, CCL7, CDKN1A, COL12A1, CTNNB1, FN1, FTH1, GSTM1, IFNG, IL4, IL6, NGF,*	*ABCB1, APC, CAT, CCNB1, CDKN2A, CYP2E1, DUSP3, EGFR, ERGIC3, GCLC, GRIK2, GSTP1, HIF1A, KEAP1, MDM2, MET, MMP9, MTOR, NFE2L2, NQO1, RAF1, SOD2, STAT3, SYP, TERT, TP53, UCHL1, VEGFA*	*CKB, EGFR, ID2, TP53, MTOR*

		PARP1, S100B, SDC1, SLC22A5, SLC2A1, TGFB2, TXN, VIM, AKT1, APOE, AR, AS3MT, CYP1B1, CYP24A1, EEF2, FOS, GJA1, GPX1, GPX3, HMOX1, HRAS, ID3, IL1B, IL2, JUNB, KDR, MAPK1, MAPK3, NOS2, OGG1, PCNA, PON1, PPP2R1B, PRKN, SELENOP, SERPINA1, SFTPB, SFTPD, SLC7A5, SMARCC1, TFRC, TGFB1, TNF, TRP53, TTR, AHCY, ALB, ANP32A, BZW2, EIF2B4, FASN, GAPDH, HK2, HNRNPA1, MAP2K1, MSH6, NCAPG, NOP56, PABPC4, RPL13A, RPL36A, RRS1, SFN, SFTPC, SNORD22		
Arsenic (As)	**10260**	*ACE, ACTB, ADA, AKT1, ANXA2, APC, APOA1, APOC3, APOE, AS3MT, ATG101, AVPI1, AZGP1, BAP1, BCL2L1, BECN1, BIRC5, BRAF, BRCA2, CA12, CALML3, CASP8, CAV1, CCL18, CCN1, CCN2, CCND1, CCNG1, CD274, CDKN1A, CDKN1B, CDKN1C, CDKN2A, CEACAM1, CHD4, CHEK2, CHST15, CLCA2, CLPTM1L, CLTB, COL6A1, COX17, CPE, CRP, CTNNB1, CWH43, CYP1A2, CYP1B1,*	*ABCB1, ADAMTS1, AKR1C1, ALK, APC, AREG, ATF3, AVEN, AXL, BIRC5, CARD18, CASP8, CAT, CCNB1, CCNH, CD74, CD9, CDKN2A, CERT1, CHFR, CLPTM1L, COL7A1, CORO1C, CST6, CXCL8, CYLD, CYP2E1, DAPK1, DUSP3, E2F1, E2F2, E2F3, E2F5, E2F6, E2F7, E2F8, EGFR, ENO1, ENO2, ERCC1, ERGIC3, FASLG, FAT1, FGF9, FHIT, FOXA1, FOXM1, GCLC, GSTM2, GSTP1, HES1, HIF1A, IL10, IL6R,*	*BIRC5, MTOR, NPPA, PYCARD, RB1, SMARCA4, TP53, UGT1A1, ASCL1, BCLAF1, CDYL, CKB, CNTNAP2, COL22A1, COL4A2, CREBBP, DIP2C, EGFR, ELAVL2, GRIK3, ID2, KHSRP, KIF21A, KIT, PTEN, RASSF8, SOX2*

		CYP24A1, CYP2E1, DAPK1, DDR1, DNASE1L3, DNMT3A, DOK2, DOK3, DPYD, EEF2, EFEMP1, EFNB2, EGFR, EGR1, EHMT2, EPHX1, ERBB2, ERBB3, ERCC1, ERCC6, ERGIC3, ESR1, FAS, FASLG, FEN1, FGF9, FHIT, FOS, FOSB, FOXM1, FOXP3, FUBP1, GATA6, GCLC, GJA1, GPX1, GPX3, GSTM1, GSTP1, GSTP2, GSTT1, HES1, HEY1, HMOX1, HNF1B, HRAS, HTRA1, ID3, IDS, IER2, IFNG, IL10, IL1B, IL1R2, IL2, IL24, IL6, IQSEC1, IRF1, IRF4, JAG1, JUN, JUNB, JUND, KDR, KRAS, LINC01089, LMNTD1, MAP2K7, MAP3K8, MAP4K4, MAPK1, MAPK14, MAPK3, MCL1, MET, MIR10A, MIR155, MIR193A, MIR21, MIR222, MIR487B, MIR494, MMP1, MPO, MTHFR, MYC, MYO18B, NFE2L2, NFYA, NOS2, NOTCH2, NPPA, NRG1, OGG1, PCNA, PDCD4, PIK3CA, PON1, PPBP, PRDX1, PRDX6, PRKN, PTEN, PTGIS, PTMA, PYCARD, RAD52, RAF1, RASSF1, RCHY1, RIOX2, ROBO1, RTEL1, RUNX3,	*ITGB1, ITGB4, KEAP1, KIF5B, KIT, KRAS, LAMC2, LGALS1, LRRC59, MACIR, MDM2, MET, MIR126, MIR205, MIR29A, MMP1, MMP11, MMP9, MS4A1, MT3, MTOR, MXRA5, MYC, NCOA3, NFE2L2, NQO1, OSMR, PHGDH, PLBD1, PRDX1, PRR13, PSAT1, PTHLH, PXN, PYCARD, RAF1, RASSF1, RB1, RBM7, RHEB, RNF168, ROS1, RRAD, SDC4, SELENOS, SLC34A2, SOCS2, SOD2, STAT3, TERT, TGFA, TMEM135, TP53, TP53BP1, UCHL1, VEGFA, XPC, ZNF462, ZSCAN31*	

		SELENBP1, SERPING1, SFTPD, SIDT2, SLC3A2, SLC67A1, SLC7A5, SLCO1B3, SMARCC1, SMC2, SND1, SOX2, SOX9, SPP1, SPRY2, STAT3, STAT5A, STK11, STN1, TERT, TFRC, TGFB1, TGFBR2, TLR4, TMEM45A, TNF, TP53, TP73, TRP53, TSC2, TTR, TYMS, USP18, VHL, WT1, XPC		
Nickel (Ni)	**7677**	*A2M, ACTB, AKT1, ANK3, ANXA2, APOA1, APOC3, APOE, AR, AS3MT, AZGP1, BECN1, BHLHE41, BIRC5, BRAF, CASP8, CCL18, CCN1, CCND1, CCNG1, CD274, CDH13, CDKN1A, CDKN1C, CDKN2A, CES1, CHEK2, CHRNA2, CHRNA3, CHRNA7, CHRNB4, CHST15, CPE, CRP, CYP1A2, CYP1B1, CYP24A1, DAPK1, DNASE1L3, DOK2, DOK3, DPYD, EAF2, EEF2, EFNB2, EGR1, EHMT2, EMX2, EPHX1, FAS, FASLG, FEN1, FGFR2, FOS, FOSB, FOSL2, FOXP3, FUBP1, GATA6, GC, GPX1, GPX3, GSTM1, GSTP2, GSTT1, HEY1, HILPDA, HMOX1, HNF1B, HTRA1, IFNG, IL10, IL1B, IL1R2, IL2, IL24, IL6, IRF1, IRF4, JUN, JUND, KCNJ15,*	*ABCB1, ADAMTS1, AREG, ATF3, BIRC5, CASP8, CAT, CCNB1, CDH13, CDKN2A, CHGA, COL7A1, CXCL8, CYLD, DAPK1, DUSP3, E2F1, E2F3, E2F7, EML4, ENO1, FASLG, GRIK2, GSTM2, HIF1A, IL10, IL6R, KEAP1, KIF5B, KIT, LRRC59, MDM2, MMP1, MMP9, MS4A1, MTOR, MYC, NCOA3, NDRG1, NEK2, NQO1, OSMR, PRDX1, PYCARD, RASSF1, RB1, RECK, SDC4, SLC34A2, SOCS2, SOD2, STAT3, TGFA, TP53, VEGFA, ZNF462, ZSCAN31*	*BIRC5, MTOR, NPPA, PYCARD, RB1, TP53, EPHA7, KIT, NDRG1, RIMS2, RUNX1T1, SATB2, ZDBF2*

		KDR, MAP4K4, MAPK1, MAPK14, MAPK3, MARCKS, MCL1, MIR154, MIR21, MIR4435-2HG, MMP1, MMP10, MPO, MTHFR, MYC, NOS2, NPPA, NRG1, OGG1, PCNA, PON1, PPBP, PRDX1, PRKN, PYCARD, RASSF1, RUNX3, SELENBP1, SELENOP, SERPINA1, SERPING1, SFTPB, SLC7A5, SMC2, SPP1, SPRY2, STAT3, STAT5A, TGFB1, TMEM45A, TNF, TP53, TSHR, TTR, TYMS, USP18, WNT5A		
Chromium(VI) (Cr(VI))	**2980**	*ACE, AKT1, APOA1, APOC3, AR, AVPI1, BCL2L1, BECN1, BIRC5, BRCA2, CASP8, CCN1, CCN2, CCND1, CCNG1, CDKN1A, CDKN1B, CDKN2A, CEACAM1, CHEK2, CHRNA5, CLCA2, COL6A1, COX17, CRP, CTNNB1, CWH43, CYP1A2, CYP1B1, CYP24A1, DPYD, EEF2, EFNB2, EGFR, EGR1, ERBB2, ERBB3, ESR1, FAS, FEN1, FGF9, FOS, FOXM1, GCLC, GPX1, GPX3, GSTM1, GSTP1, HILPDA, HMOX1, HRAS, HTRA1, IDS, IER2, IFNG, IL1B, IL2, IL24, IL6, IQSEC1, IRF1, JUN, JUNB, LECT2, MAP2K7, MAPK1,*	*ADAMTS1, AREG, ATF3, BIRC5, CASP8, CAT, CCNB1, CCNH, CDKN2A, CXCL8, E2F1, E2F2, E2F8, EGFR, ENO2, FAT1, FGF9, FOXM1, FOXO3, GCLC, GSTP1, HIF1A, ITGB4, KDM1A, KIT, MIR625, MMP1, MMP9, MTOR, MXRA5, MYC, NDRG1, NEK2, NFE2L2, NQO1, OSMR, OXTR, PHLDA2, PPIA, PRDX1, PSAT1, PYCARD, RASSF1, RB1, ROS1, RRAD, RRM1, SHMT2, SOD2, STAT3, TERT, TGFA, TMEM135, TP53, TP53BP1, VEGFA*	*BIRC5, MTOR, PYCARD, RB1, TP53, COL4A2, CREBBP, EGFR, ELAVL2, EP300, KIT, NDRG1, RUNX1T1, SLIT2, SOX2*

		MAPK14, MAPK3, MIR21, MIR302D, MIR494, MIR607, MMP1, MMP10, MPO, MYC, NFE2L2, NOS2, OGG1, PCNA, PDCD4, PRDX1, PRDX6, PRKN, PTMA, PYCARD, RASSF1, SERPINA1, SERPING1, SFTPB, SIDT2, SLC7A5, SMC2, SOX2, SOX9, SPRY2, STAT3, TERT, TFRC, TGFBR2, TNF, TP53, TRP53, TYMS, USP18, WNT5A		
Pb+Cd+MMC+As+Ni+Cr(VI)		*AKT1, CDKN1A, FOS, GPX1, GPX3, GSTM1, HMOX1, IFNG, IL1B, IL6, MAPK1, MAPK3, NOS2, OGG1, PRKN, STAT3, TNF,* ***TP53***	*CAT, CCNB1, MTOR, NQO1, SOD2, STAT3,* ***TP53****, VEGFA*	*MTOR,* ***TP53***

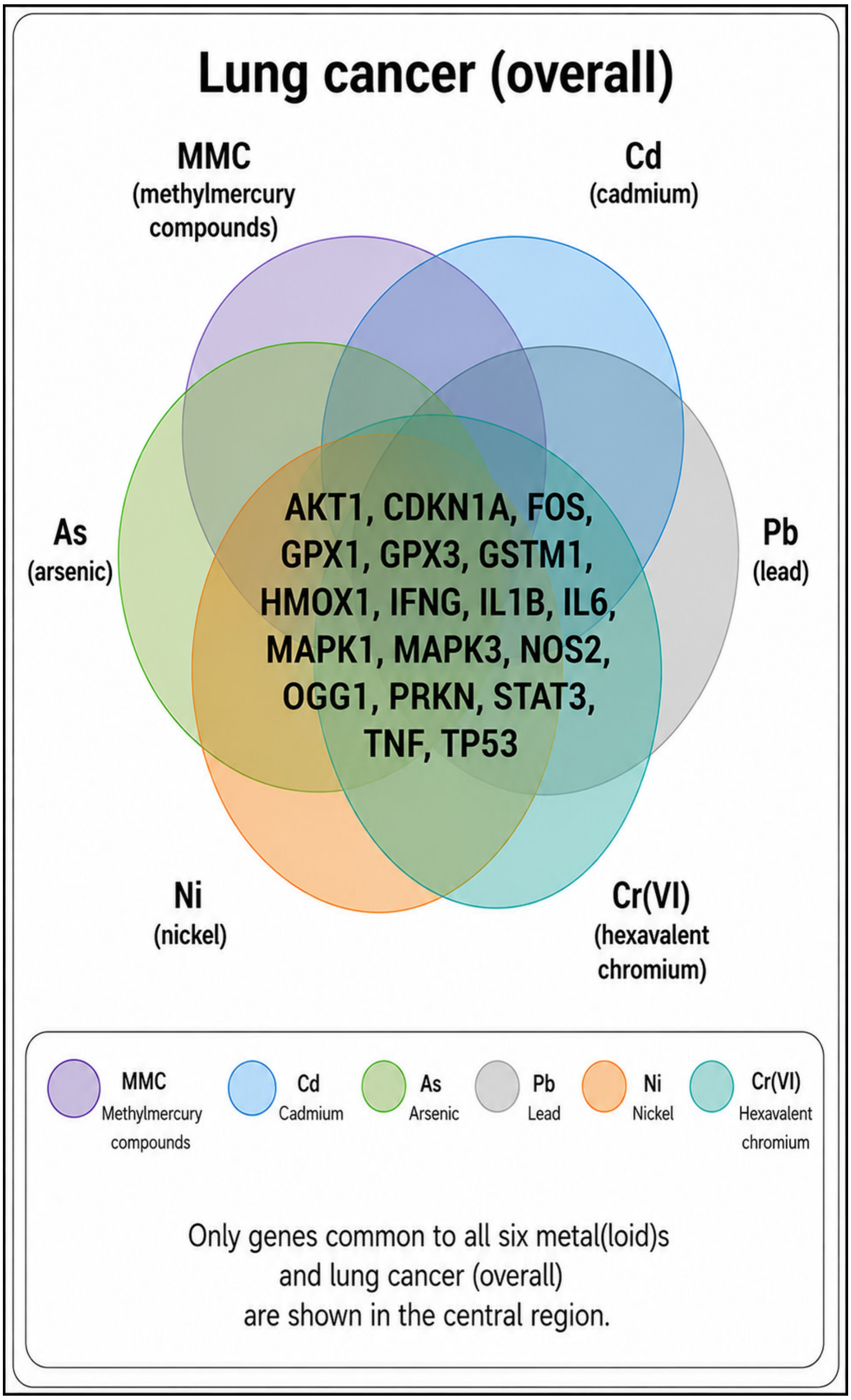

Figure S1. Common target genes associated with Pb, Cd, MMC, As, Ni and Cr(VI) exposure and lung cancer.

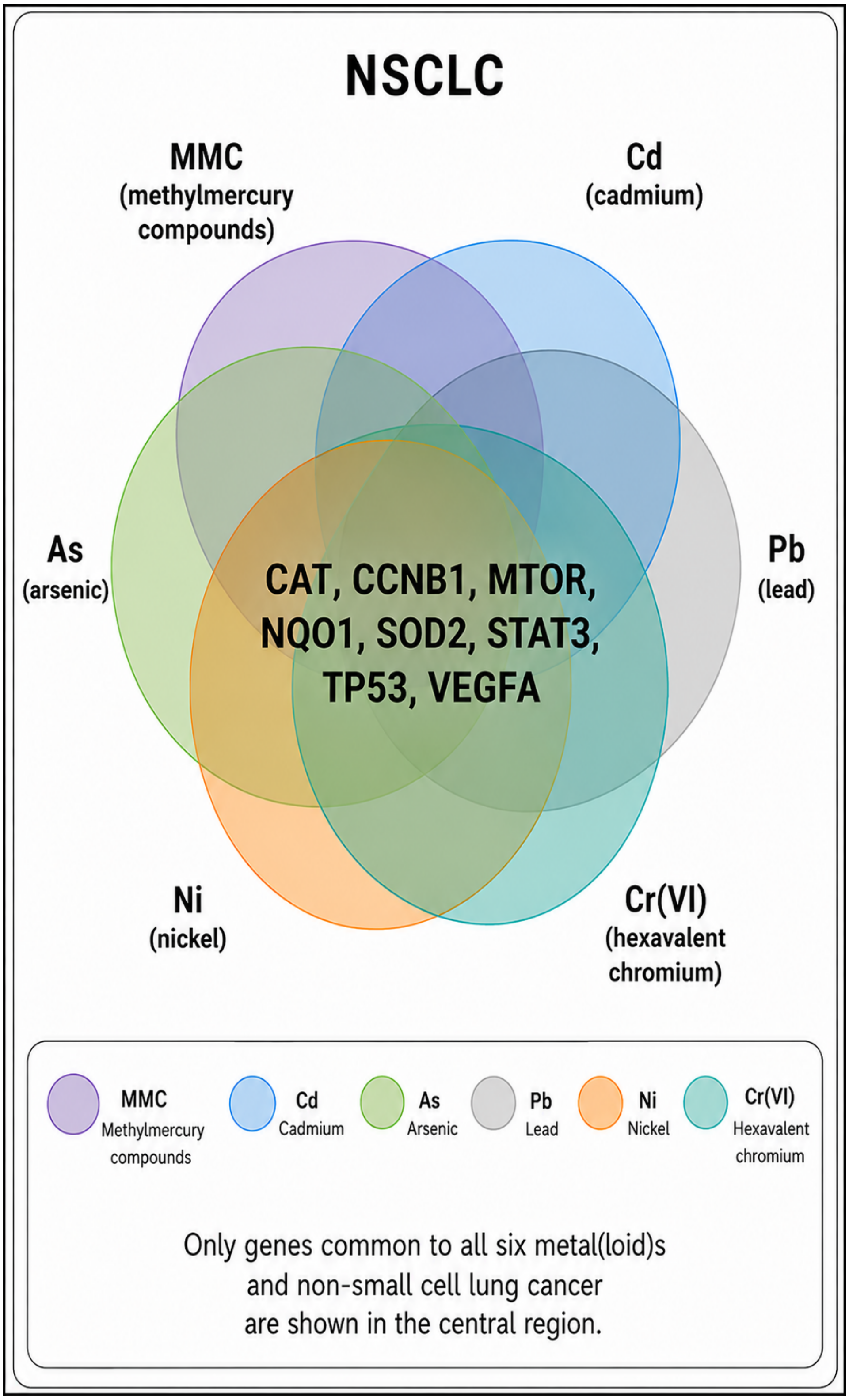

Figure S2. Common target genes associated with Pb, Cd, MMC, As, Ni and Cr(VI) exposure and NSCLC.

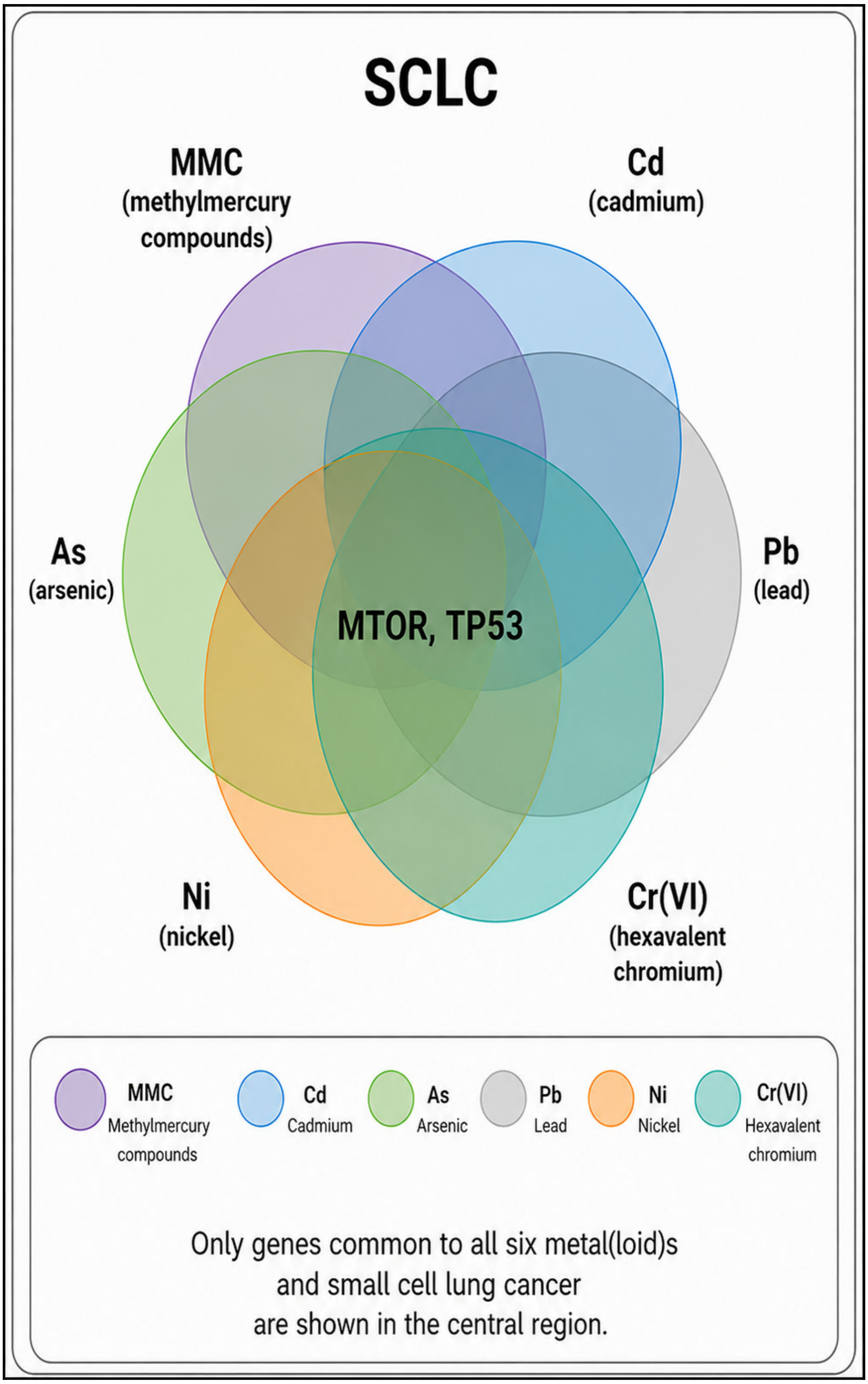

Figure S3. Common target genes associated with Pb, Cd, MMC, As, Ni and Cr(VI) exposure and SCLC.

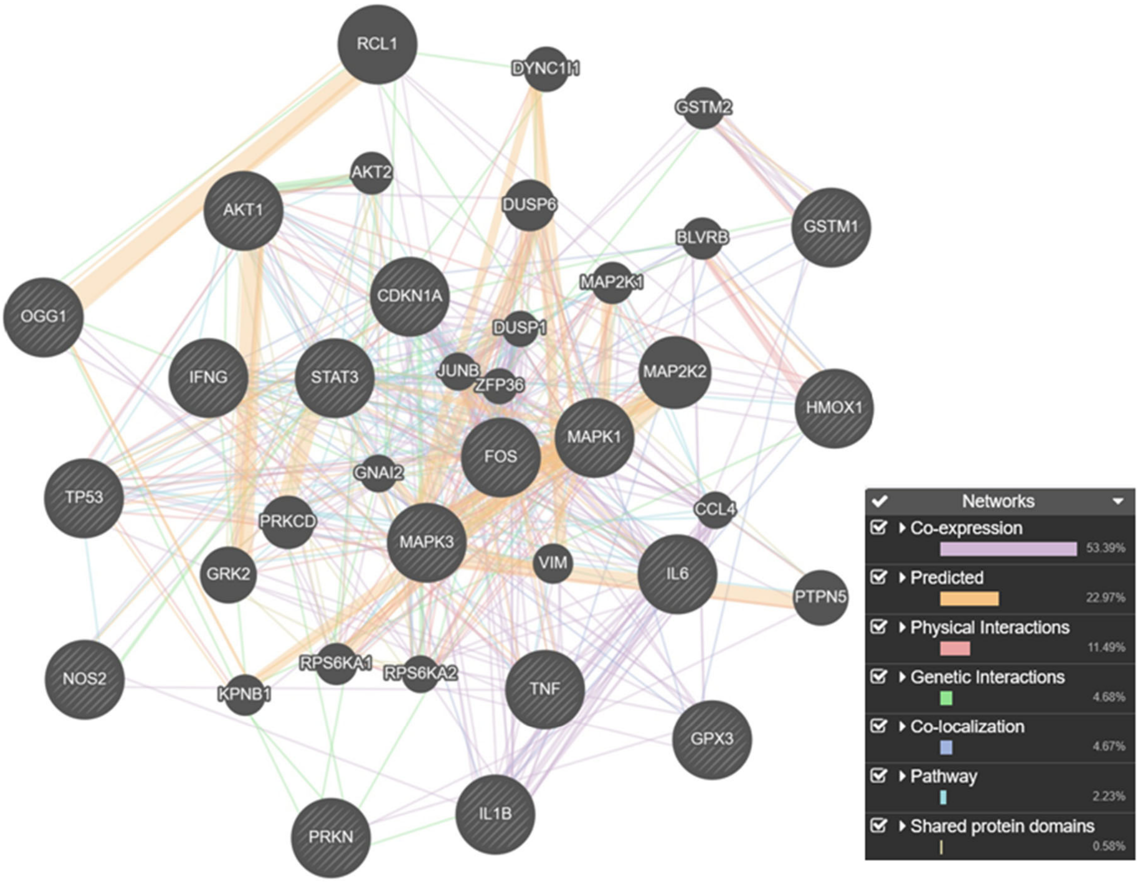

Figure S4. Interactions between target genes linked with Pb, Cd, MMC, As, Ni and Cr (VI) exposure and lung cancer, together with 20 related genes. The target genes common to the investigated toxic metal(loid) are represented as striped nodes (circles with diagonal line) while circles without diagonal line indicate related genes. The size of the predicted nodes (additional genes predicted by GeneMANIA) reflects computed degree of association (degree to which they are predicted to be related) to the seed nodes, with largest genes representing the most reliable (higher confidence) interactors.

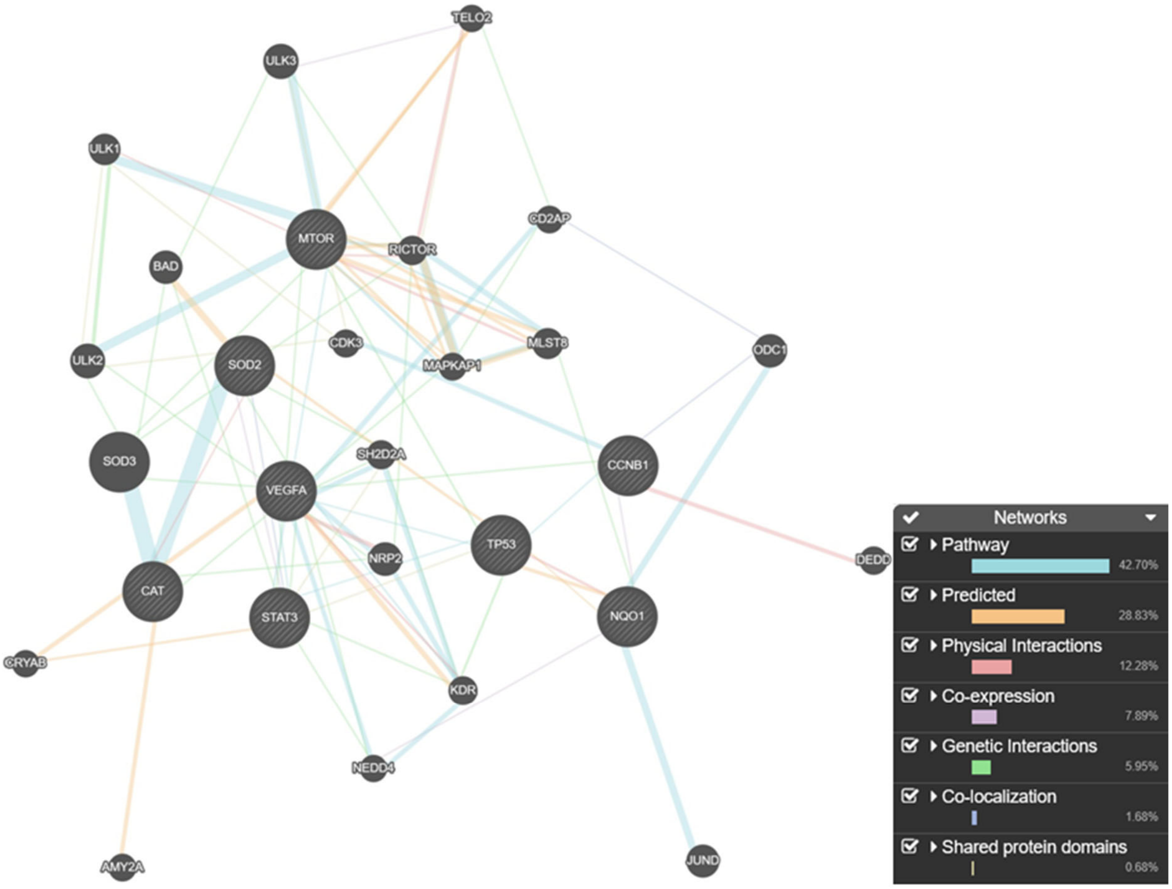

Figure S5. Interactions between target genes linked with Pb, Cd, MMC, As, Ni and Cr (VI) exposure and NSCLC, together with 20 related genes. The target genes common to the investigated toxic metal(loid) are represented as striped nodes (circles with diagonal line) while circles without diagonal line indicate related genes. The size of the predicted nodes (additional genes predicted by GeneMANIA) reflects computed degree of association (degree to which they are predicted to be related) to the seed nodes, with largest genes representing the most reliable (higher confidence) interactors.

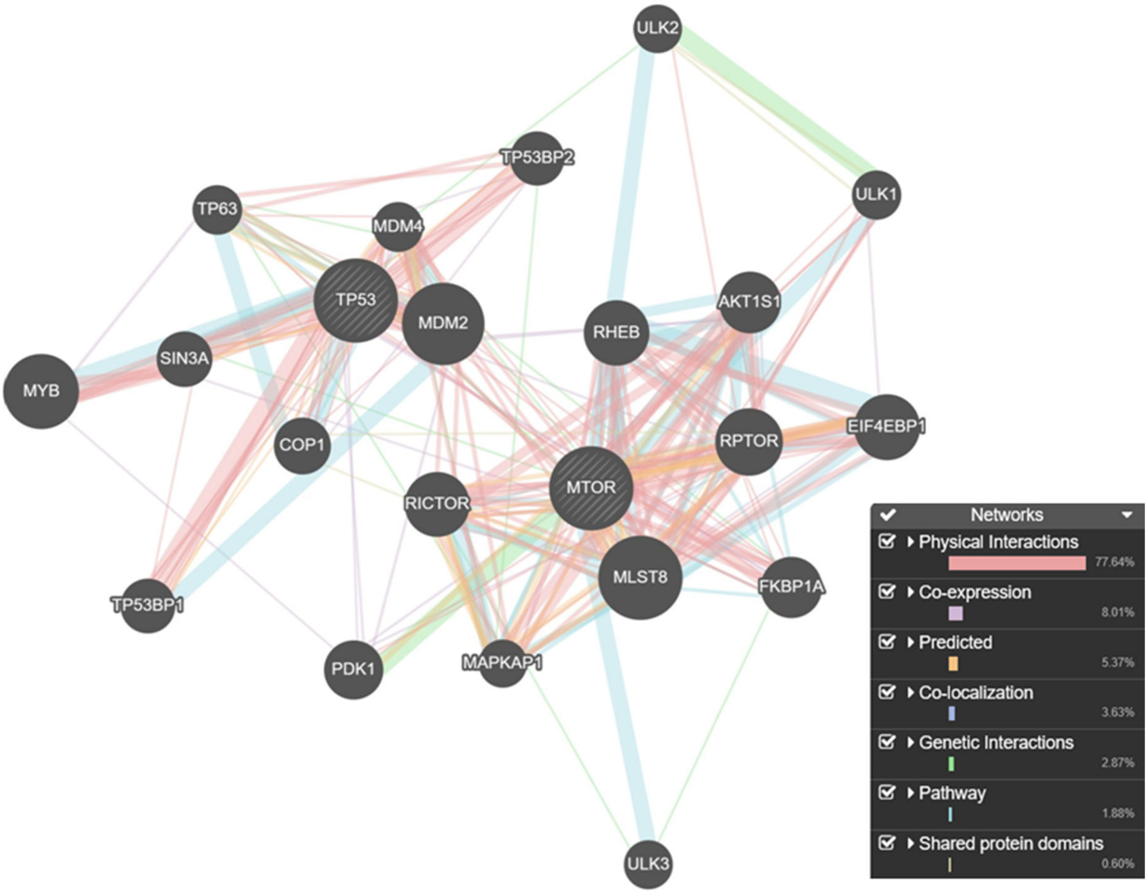

Figure S6. Interactions between target genes linked with Pb, Cd, MMC, As, Ni and Cr (VI) exposure and SCLC, together with 20 related genes. The target genes common to the investigated toxic metal(loid) are represented as striped nodes (circles with diagonal line) while circles without diagonal line indicate related genes. The size of the predicted nodes (additional genes predicted by GeneMANIA) reflects computed degree of association (degree to which they are predicted to be related) to the seed nodes, with largest genes representing the most reliable (higher confidence) interactors.

Table S2. Enriched signaling pathways identified using genes shared across the investigated toxic metal(loid)s (Pb, Cd, MMC, As, Ni, and Cr(VI)) and lung cancer, together with their 20 related genes identified by GeneMANIA (based on CTD, GeneMANIA and ToppFun tool).

**Pathway name**	**Term ID**	**p-value**	**FDR-adjusted p-value**	**Genes**
REACTOME SIGNALING BY INTERLEUKINS	M874	5.637E-22	1.123E-18	*CDKN1A, PTPN5, VIM, JUNB, AKT1, CCL4, TNF, HMOX1, MAPK1, MAPK3, IL1B, MAP2K1, NOS2, IL6, FOS, RPS6KA1, RPS6KA2, TP53, STAT3, DUSP6*
REACTOME CYTOKINE SIGNALING IN IMMUNE SYSTEM	M1060	2.016E-21	2.008E-18	*IFNG, CDKN1A, PTPN5, VIM, PRKCD, JUNB, AKT1, CCL4, AKT2, TNF, HMOX1, MAPK1, MAPK3, IL1B, MAP2K1, NOS2, IL6, FOS, RPS6KA1, RPS6KA2, TP53, STAT3, DUSP6, KPNB1*
WP MACROPHAGESTIMULATING PROTEIN MSP SIGNALING	M48078	7.178E-21	4.767E-18	*VIM, AKT1, TNF, MAPK1, MAPK3, IL1B, MAP2K1, MAP2K2, IL6, FOS, RPS6KA1, DUSP1, TP53, DUSP6*
WP MAPK SIGNALING PATHWAY	MM15990	2.177E-18	1.084E-15	*PTPN5, PRKCD, AKT1, AKT2, TNF, MAPK1, MAPK3, IL1B, MAP2K1, MAP2K2, FOS, DUSP1, TP53, DUSP6*
REACTOME INTERLEUKIN 4 AND INTERLEUKIN 13 SIGNALING	M27609	6.199E-17	2.470E-14	*CDKN1A, VIM, JUNB, AKT1, TNF, HMOX1, IL1B, NOS2, IL6, FOS, TP53, STAT3*

P-values were adjusted using the Benjamini–Hochberg (BH) False Discovery Rate (FDR) procedure, with significance defined as FDR-adjusted $p < 0.05$.

Table S3. Enriched signaling pathways identified using genes shared across the investigated toxic metal(loid)s (Pb, Cd, MMC, As, Ni, and Cr(VI)) and NSCLC, together with their 20 related genes identified by GeneMANIA (based on CTD, GeneMANIA and ToppFun tool).

Pathway name	Term ID	p-value	FDR-adjusted p-value	Genes
WP TARGET OF RAPAMYCIN SIGNALING	M39635	4.027E-13	4.334E-10	*MAPKAP1, ULK3, RICTOR, ULK1, MLST8, ULK2, MTOR*
KEGG MTOR SIGNALING PATHWAY	M7561	6.322E-12	3.401E-9	*ULK3, RICTOR, ULK1, MLST8, ULK2, MTOR, VEGFA*
REACTOME SIGNALING BY VEGF	MM14752	1.559E-11	5.299E-9	*MAPKAP1, RICTOR, KDR, SH2D2A, MLST8, MTOR, NRP2, VEGFA*
REACTOME SIGNALING BY VEGF	M27077	1.970E-11	5.299E-9	*MAPKAP1, RICTOR, KDR, SH2D2A, MLST8, MTOR, NRP2, VEGFA*
WP PI3K-AKT-MTOR SIGNALING AND THERAPEUTIC OPPORTUNITIES IN PROSTATE CANCER	M39445	2.839E-11	6.110E-9	*MAPKAP1, RICTOR, ULK1, MLST8, MTOR, BAD*

P-values were adjusted using the Benjamini–Hochberg (BH) False Discovery Rate (FDR) procedure, with significance defined as FDR-adjusted $p < 0.05$.

Table S4. Enriched signaling pathways identified using genes shared across the investigated toxic metal(loid)s (Pb, Cd, MMC, As, Ni, and Cr(VI)) and SCLC, together with their 20 related genes identified by GeneMANIA (based on CTD, GeneMANIA and ToppFun tool).

Pathway name	Term ID	p-value	FDR-adjusted p-value	Genes
WP TARGET OF RAPAMYCIN SIGNALING	M39635	5.470E-27	3.851E-24	*MAPKAP1, ULK3, FKBP1A, ULK2, MTOR, AKT1S1, RICTOR, RPTOR, ULK1, RHEB, EIF4EBP1, MLST8*
WP PI3K-AKT-MTOR SIGNALING AND THERAPEUTIC OPPORTUNITIES IN PROSTATE CANCER	M39445	2.056E-19	7.236E-17	*MAPKAP1, MTOR, PDK1, RICTOR, RPTOR, ULK1, RHEB, EIF4EBP1, MLST8*
PID MTOR 4PATHWAY	M121	2.216E-18	5.199E-16	*MAPKAP1, ULK2, MTOR, AKT1S1, RICTOR, RPTOR, ULK1, RHEB, EIF4EBP1, MLST8*
WP HEAD AND NECK SQUAMOUS CELL CANCER	M39857	4.663E-18	8.207E-16	*MAPKAP1, FKBP1A, MTOR, RICTOR, RPTOR, TP63, TP53, RHEB, EIF4EBP1, MLST8*
KEGG MTOR SIGNALING PATHWAY	M7561	2.652E-17	3.734E-15	*ULK3, ULK2, MTOR, RICTOR, RPTOR, ULK1, RHEB, EIF4EBP1, MLST8*

P-values were adjusted using the Benjamini–Hochberg (BH) False Discovery Rate (FDR) procedure, with significance defined as FDR-adjusted $p < 0.05$.
